# Effects of preventive interventions on neuroimaging biomarkers in subjects at-risk to develop Alzheimer's disease: A systematic review

**DOI:** 10.3389/fnagi.2022.1014559

**Published:** 2022-11-24

**Authors:** Lisa Perus, Germain U. Busto, Jean-François Mangin, Emmanuelle Le Bars, Audrey Gabelle

**Affiliations:** ^1^INM, Univ Montpellier, INSERM, CHU Montpellier, Montpellier, France; ^2^Department of Neurology, Memory Resources and Research Center, Gui de Chauliac Hospital, Montpellier, France; ^3^Institut d'Imagerie Fonctionnelle Humaine, I2FH, Department of Neuroradiology, Gui de Chauliac Hospital and University of Montpellier, Montpellier, France; ^4^CATI, US52-UAR2031, CEA, ICM, SU, CNRS, INSERM, APHP, Ile de France, France; ^5^Université Paris-Saclay, CEA, CNRS, Neurospin, UMR9027 Baobab, Gif-sur-Yvette, France

**Keywords:** nutrition, cognitive training, physical exercise, multidomain intervention, brain imaging, Alzheimer's disease, prevention, biomarkers

## Abstract

Alzheimer's Disease (AD) is a multifactorial and complex neurodegenerative disorder. Some modifiable risk factors have been associated with an increased risk of appearance of the disease and/or cognitive decline. Preventive clinical trials aiming at reducing one or combined risk factors have been implemented and their potential effects assessed on cognitive trajectories and on AD biomarkers. However, the effect of interventions on surrogate markers, in particular imaging biomarkers, remains poorly understood. We conducted a review of the literature and analyzed 43 interventional studies that included physical exercise, nutrition, cognitive training or multidomain interventions, and assessed various brain imaging biomarkers, to determine the effects of preventive interventions on imaging biomarkers for subjects at-risk to develop AD. Deciphering the global and regional brain effect of each and combined interventions will help to better understand the interplay relationship between multimodal interventions, cognition, surrogate brain markers, and to better design primary and secondary outcomes for future preventive clinical trials. Those studies were pondered using generally-admitted quality criteria to reveal that interventions may affect the brain of patients with cognitive impairment rather than those without cognitive impairment thus indicating that particular care should be taken when selecting individuals for interventions. Additionally, a majority of the studies concurred on the effect of the interventions and particularly onto the frontal brain areas.

## 1. Introduction

Alzheimer's disease (AD) is a devastating neurodegenerative disorder characterized by a complex and multifactorial physiopathology. Clinically, the typical form induces episodic memory deficit, progressively associated with language and behavioral troubles and leading to a loss of autonomy (DeTure and Dickson, 2019; Breijyeh and Karaman, 2020; Scheltens et al., 2021). Brain amyloidosis and neurodegenerative processes remain the main therapeutic targets as they occur many years prior to cognitive and clinical symptoms appearance (Sperling et al., 2013; Makin, 2018).

Two interventional strategies have been developed, one focusing on drugs targeting specific molecules such as the amyloid-beta (Aβ) peptide or Tau protein, and the other focusing on holistic non-specific targets such as epidemiological and/or and exposome risk factors. For the first strategy, Aduhelm has recently been FDA-approved for US AD patients as a specific anti-amyloid drug (Cummings et al., 2021). The readout for other anti-amyloid phase 3 drugs are coming and other Tau, neuroinflammation and APOE ε4 targets are studied. For the second category, multimodal preventive interventions are promoted by governmental health organizations (WHO) based on epidemiological and interventional clinical trials data (World Health Organization, 2019). Modifiable risk factors such as low education, midlife hypertension, midlife obesity, diabetes, physical activity (PA), smoking or depression have been linked to AD (Norton et al., 2014; Serrano-Pozo and Growdon, 2019). By acting on those risk factors before the apparition of clinical symptoms, one third of AD cases could be potentially reduced with reasonable costs (Livingston et al., 2017).

Healthy lifestyles (PA, nutrition, cognitive stimulation…) are associated with lower incidence of AD (Dhana et al., 2020). The amount of PA has clearly been inversely associated with the risk of cognitive decline and AD (Paillard, 2015). Aerobic physical exercise (PE) induces the release of neurotrophic factors and reduces the production of free radicals, both phenomena participating in improving memory and cognitive function while limiting the alteration of specific neuronal populations (Paillard et al., 2015). Nutrition and diet might be significant modifiable risk factors of AD and multiple antioxydants, vitamins, polyphenols, fish, or dietary patterns (Japanese, Mediterranean) have been reported to decrease the risk of AD (Hu et al., 2013). Cognitive training (CT) seems also very promising (Sitzer et al., 2006) by targeting several domains of cognition such as memory, executive or visuospatial functions (Nguyen et al., 2019). While preventive multimodal interventions for AD including PE (Erickson et al., 2011; Zhu et al., 2020; López-Ortiz et al., 2021), nutrition/diet (Cremonini et al., 2019) or CT (Buschert et al., 2010) have shown promising results, the majority of these trials have small sample sizes and evidences from large single-domain lifestyle interventions (PE, LIFE study [Longitudinal Impact of Fitness and Exercise]; dietary, OPAL study [Older People And n-3 Long-chain polyunsaturated fatty acid]; CT, ACTIVE [Advanced Cognitive Training in Vital Elderly], IHAMS [Iowa Healthy and Active Minds Study]) are limited (Kivipelto et al., 2018).

As AD is multifactorial, multi-domains (MD) interventions would be more relevant than individual factors or even have a superadditive effect on clinically meaningful outcomes (Coley et al., 2008; Scarmeas, 2009; Kivipelto et al., 2018). The combination of interventions has been addressed by clinical trials (FINGER, MAPT, PreDIVA; Richard et al., 2009; Vellas et al., 2014; Ngandu et al., 2015), and different hypotheses have been made about the potential synergistic effects between interventions. For instance, PE could increase the potential for neuro-, synapto-, and angiogenesis while CT would guide it to the stimulated brain regions (Bamidis et al., 2014). Nutrition, including omega-3 (ω3) intake, could fuel structural changes associated with these interventions (Köbe et al., 2016). Interestingly, multimodal interventions may be more effective before clinical symptoms, especially for at-risk of AD populations such as the carriers of the ϵ4 allele of the apolipoprotein E (APOE ϵ4; Berkowitz et al., 2018). The primary outcomes to define the efficacy of these interventions are on cognitive performances. The effect on surrogate biomarkers is less described or as exploratory analyses (Rolandi et al., 2016).

Thus, we aimed to evaluate the effect of multimodal interventions alone or combined such as PE, CT, and nutrition/diet on a large variety of brain imaging outcomes analyzed globally and regionally in participants that may develop AD. We also assessed whether this effect may be dependent on the cognitive status of the population included in the studies.

## 2. Materials and methods

### 2.1. Studies selection

A search of the PUBMED database was performed on May the 17th of 2021. The query included the following terms: (“elderly” OR “frail elderly” OR “risk factors” OR “MCI” OR “alzheimer”) AND (“PET” OR “brain imaging” OR “MRI” OR “structural MRI” OR “functional MRI”) AND (“training” OR “nutrition” OR “diet” OR “physical activity” OR “cognitive training” OR “cognitive stimulation” OR “exercise”). The full search strategy is provided in the Supplementary material. We selected preventive studies involving older adults susceptible to convert to AD and evaluated the effect of CT, nutrition/diet, PE or MD interventions on brain imaging outcomes. The description of the aim of this review, using the PICO framework (Huang et al., 2006), is available in Supplementary Table 1. As our goal was to examine the effect of interventions on brain regions, including areas located in subcortical structure, we did not select studies using electrophysiological techniques such as electroencephalography (EEG), which have a relatively low spatial resolution (Krishnaswamy et al., 2017). Older adults were considered susceptible to convert to AD if the studies in which they were included stated that they exhibited risk factors for AD (e.g., APOE ϵ4) and/or cognitive impairments (subjective or objective) and/or biological biomarkers of AD (e.g., elevated amyloid load). The participants at risk for AD were defined into two groups with either no objective cognitive impairment (nCI) or objective cognitive impairment (CI). The nCI category groups together different type of participants: participants with risk factors for AD such as hypertension are included in this category, as well as participants with subjective cognitive decline. For the participants with CI, they could also present positive neuroimaging or cerebrospinal fluid (CSF) biomarkers.

We excluded (1) observational studies, (2) studies including exclusively healthy older adults (HOA) not predisposed to conversion to AD, (3) studies including exclusively patients already diagnosed with AD, (4) studies including populations with additional neurodegenerative diseases or vascular cognitive impairment, (5) articles not written in English, (6) studies for which only the abstract was available. We did not include yoga and dance interventions as we consider them special types of exercises. Yoga is a spiritual activity that encompasses physical exercise, controlled breathing, and meditation training. Dance is an artistic expression requiring memorization and execution of a series of movements according to the rhythm of a type of music and to the movements of a partner. The brain processes involved in these activities may be different from those involved in a simpler form of physical exercise. It has indeed been suggested that yoga and dance could have different effects on the brain from traditional forms of physical exercise (Rehfeld et al., 2018; van Aalst et al., 2021; Kaur et al., 2022). Analysis was not restricted to studies including a control for the interventions (i.e., a “placebo” or “sham” intervention), and no additional restriction was applied to the control condition when included (i.e., for two studies evaluating the effect of PE, control condition could either be resistance or balance training). When multiple brain imaging outcomes were evaluated in a study, they were all reported and equally considered. The distinct types of brain imaging data are referred to as imaging “modalities.” Article's abstract screening was performed by LP and full-text review was performed by GB and LP independently and validated by AG and ELB. Any discrepancy was resolved through discussion until a consensus was obtained. The inclusion of the studies was described by a PRISMA flowchart generated by the PRISMA 2020 Shiny application (https://estech.shinyapps.io/prisma_flowdiagram/; Haddaway et al., 2022).

### 2.2. Criteria used to assess the quality of selected studies

Multiple criteria were used to evaluate and compare the relative quality of the studies included. Part of these criteria are listed as the first thirteen items of Table 1 and were previously defined by Pitkälä et al. (2013), and used in a review specifically focussed on brain imaging outcomes (Haeger et al., 2019). To fit with the specificity of this review, we added/adapted some criteria. First, studies without a sham group were included and, in those cases, criteria #6 and #9 were applied to the comparisons between types of populations [e.g., HOA vs. mild cognitive impairment (MCI); Table 1]. Second, for studies evaluating only imaging outcomes, blinding criterion (#10) was not taken into consideration. Third, criterion concerning studies' power (#4) was reconsidered acknowledging a recent review on neuroimaging studies: in 2017–2018, only 3–4% of them did an a priori power calculation (Szucs and Ioannidis, 2020). As most intervention trials are expected to have at least two groups (intervention and sham), we estimated that studies were sufficiently powered if a proper power calculation was made or if there were at least 20 participants per group—considering that 11–56% of clinical studies with a single group and published between 1990–2012 had at least 40 participants (Szucs and Ioannidis, 2020). Study power was assessed as part of the overall quality assessment. However, power itself was not a criterion for exclusion. All studies were examined and included in this review, regardless of whether they were sufficiently powered or not. Additional quality criteria specific to neuroimaging studies were assessed: (1) imaging protocol and analyses had to be adequately described and (2) appropriate correction for multiple testing had to be implemented (e.g., voxel-wise analysis). An additional point was attributed to studies performing extra quality controls for raw imaging data or analyses' outputs. All criteria were assessed independently by GB and LP, differences in notation were discussed until a consensus was met.

**Table 1:** Notation criteria used to rank studies' methodology.

1. The study is a randomized controlled trial with an adequate method of randomization
2. The studied population is well-defined
3. Inclusion and exclusion criteria are adequately described
4. The study is sufficiently powered to detect an effect of the intervention (the number of participants is justified by a power calculation or seems adequate)
5. Outcomes measures are valid and clearly defined
6. Groups are comparable at baseline or outcomes measures are adjusted as needed
7. Drop-outs are described (e.g., with a flowchart of participants exclusion) and taken into account in the analyses
8. Analysis is performed on an intention-to-treat basis
9. A comparison on the change of the outcome variable is done between the groups
10. Blinding to group assignment when evaluating the outcomes
11. The intervention is aptly described
12. The compliance of participants is reported
13. Complications are described
14. Imaging protocols and analyses are adequately described
15. If needed, methods are applied to correct for multiple testing on brain imaging data
16. Quality control procedures are implemented

Studies were attributed a percentage of validated criteria and classified into limited, good and high quality if they respectively had < 50%, between 50 and 80% and more than 80% of validated items.

### 2.3. Criteria to assess the different types of results according to cognitive profile

The effects of interventions on neuroimaging modalities was assessed for nCI and CI participants (Supplementary Figure 1). For cases with multiple modalities tested, the effect was considered for each type. We define “k” as the number of “results” for all modalities and for ***N*** studies (a result relates to either the effect (positive result) or lack of effect (null result) of an intervention for one modality). We did not take into account results on pooled participants (e.g., mixed population of MCI and HOA), unless a distinction was made between the populations. Similarly, we discussed separately results associated with sub-analyses for specific subgroups (e.g., participants with APOE ϵ4 status), or that were reflecting correlations. Measures obtained at intermediate time-points during the intervention or long-term follow-up measures were discussed separately. Only measures obtained directly after the end of the intervention were examined.

A score (called “s”) quantifying the effect of interventions for each neuroimaging modality on participants was computed as:


(1) $$\begin{array}{l} s=\underset{x\in E}{\sum }\left(control\left(x\right)+quality\left(x\right)\right)-\underset{x\in A}{\sum }\left(control\left(x\right)+quality\left(x\right)\right) \end{array}$$

where E were the results reporting an effect of intervention on neuroimaging biomarkers and A was the set of results associated with an absence of effect of the interventions. Control and quality functions were defined as:


(2) $$control(x)=\{\begin{array}{ll} 1 & \text{controlcondition},\\ 0 & \text{nocontrolcondition}. \end{array}$$

and


(3) $$quality(x)=\{\begin{array}{ll} 1 & q<50\%,\\ 2 & 50\%\leq q\leq 80\%,\\ 3 & 80\%<q. \end{array}$$

where *q* was the percentage of items validated in the result study quality notation (see Table 2).

**Table 2:** Assessment of studies' methodology (refer to Table 1 for the description of the notation criteria).

**References**	**#1**	**#2**	**#3**	**#4**	**#5**	**#6**	**#7**	**#8**	**#9**	**#10**	**#11**	**#12**	**#13**	**#14**	**#15**	**#16**	**Note (% of items validated)**
**Physical exercise**																	
Vidoni et al. (2021)	+	+	+	+	+	+	+	+	+	+	+	+	+	+	NA	−	14.0 (93%)
Tarumi et al. (2020)	+	+	+	−	+	+	+	−	+	+	+	+	+	+	+	+	14.0 (88%)
ten Brinke et al. (2015)	+	+	+	−	+	+	+	+	+	+	+	+	+	+	−	+	14.0 (88%)
Tomoto et al. (2021)	+	+	+	−	+	+	+	+	+	+	+	+	±	+	NA	−	12.0 (80%)
Yogev-Seligmann et al. (2021)	+	+	+	−	+	+	±	−	+	−	+	+	+	+	+	+	12.0 (75%)
Kaufman et al. (2021)	+	+	+	+	+	+	±	−	+	+	+	+	−	+	NA	−	11.0 (73%)
Thomas et al. (2020)	+	+	+	−	+	+	±	−	+	+	+	+	+	+	±	−	11.0 (69%)
Alfini et al. (2019)	−	+	+	−	+	+	±	−	+	+	+	+	±	+	+	+	11.0 (69%)
Smith et al. (2013)	−	+	+	−	+	+	±	−	+	+	+	+	±	+	+	−	10.0 (62%)
Chirles et al. (2017)	−	+	+	−	+	+	−	−	+	+	+	+	−	+	+	−	10.0 (62%)
Henrique de Gobbi Porto et al. (2015)	−	+	+	+	+	±	±	−	+	−	+	+	+	±	±	−	8.0 (50%)
**Nutrition**																	
Soininen et al. (2017)	+	+	+	+	+	+	+	−	+	+	+	+	+	+	NA	+	14.0 (93%)
Soininen et al. (2021)	+	+	+	+	+	+	+	−	+	+	+	+	+	+	NA	+	14.0 (93%)
Smith et al. (2010)	+	+	+	+	+	±	+	+	+	+	+	+	+	+	NA	−	13.0 (87%)
Zhang et al. (2016)	+	+	+	+	+	+	±	+	+	+	+	+	−	±	−	−	11.0 (69%)
Köbe et al. (2017)	+	+	+	-	+	±	±	−	+	+	+	+	+	+	±	+	11.0 (69%)
Jernerén et al. (2015)	+	+	+	+	+	±	±	−	+	NA	+	+	±	+	NA	−	9.0 (64%)
Neth et al. (2020)	+	+	+	−	+	+	±	−	±	±	+	+	+	+	+	−	10.0 (62%)
Schwarz et al. (2017)	±	+	+	−	+	±	±	−	+	NA	+	+	±	+	NA	−	7.0 (50%)
Hama et al. (2020)	−	±	±	+	+	NA	±	−	NA	+	±	−	−	+	NA	−	4.0 (31%)
Manzano Palomo et al. (2019)	−	+	±	−	+	±	±	−	+	−	−	±	−	−	NA	−	3.0 (20%)
**Cognitive training**																	
Simon et al. (2018)	±	+	+	−	+	+	+	+	+	±	+	−	−	+	+	+	11.0 (69%)
Li et al. (2019)	±	+	+	+	+	−	±	−	+	+	+	±	+	+	+	+	11.0 (69%)
Barban et al. (2017)	+	+	+	−	+	+	±	−	+	+	+	−	−	+	+	+	11.0 (69%)
Ciarmiello et al. (2015)	±	+	+	−	+	+	+	+	+	−	+	−	−	+	+	−	10.0 (62%)
Youn et al. (2019)	+	+	+	−	+	+	±	−	+	+	+	−	±	+	+	−	10.0 (62%)
Belleville et al. (2011)	−	+	+	−	+	+	+	+	+	+	+	−	−	+	−	−	10.0 (62%)
Zhang et al. (2019)	−	+	+	−	+	NA	±	−	NA	+	+	+	−	+	+	±	8.0 (57%)
Park et al. (2019)	+	+	+	−	+	+	+	−	+	±	+	±	±	+	−	−	9.0 (56%)
Yang et al. (2016)	±	+	+	−	+	+	±	−	+	−	+	−	±	+	−	+	8.0 (50%)
Na et al. (2018)	−	+	+	−	+	±	+	+	−	−	+	+	−	+	−	−	8.0 (50%)
Vermeij et al. (2016)	−	+	+	−	+	+	±	−	+	−	+	±	−	+	−	+	8.0 (50%)
Feng et al. (2018)	−	+	+	−	±	+	±	−	+	+	+	−	−	±	+	−	7.0 (44%)
Hohenfeld et al. (2017)	−	+	+	−	+	±	−	−	+	−	+	±	−	+	+	−	7.0 (44%)
**Multidomain intervention**																	
Train the Brain Consortium (2017)	+	+	+	+	+	+	+	+	+	+	+	±	+	+	+	−	14.0 (88%)
van Dalen et al. (2017)	+	+	+	+	+	±	+	+	+	NA	+	+	+	+	NA	±	12.0 (86%)
Stephen et al. (2019)	+	+	+	+	+	+	±	−	+	+	+	−	+	+	+	+	13.0 (81%)
Delrieu et al. (2020)	+	+	+	+	+	+	±	+	+	+	+	+	−	+	+	−	13.0 (81%)
Broadhouse et al. (2020)	+	+	+	+	+	±	±	−	+	+	+	−	−	+	+	+	11.0 (69%)
Stephen et al. (2020)	+	+	+	+	+	+	±	−	+	+	+	−	±	+	+	−	11.0 (69%)
Köbe et al. (2016)	±	+	+	−	+	+	+	−	+	−	+	+	−	+	+	−	10.0 (62%)
Anderson-Hanley et al. (2018)	+	±	±	−	+	+	±	−	+	−	+	+	+	+	−	+	9.0 (56%)
Fotuhi et al. (2016)	−	+	+	−	±	NA	−	−	NA	−	+	−	−	−	NA	−	3.0 (23%)

Quality criteria : #1 : adequate randomization, #2 well-defined population, #3 : well-described inclusion/exclusion criteria, #4 : statistical power, #5 : clear outcome, #6 : groups are comparable, #7 : drop-out described, #8 : intention-to-treat, #9 : between-group comparisons, #10 : blinding for outcome evaluation, #11 : well-described intervention, #12 : description of compliance, #13 description of complications, #14 : clear neuroimaging protocols/analyses, #15 : correction for multiple testing, #16 : quality control procedures. NA, criteria not applicable.

### 2.4. Effects of interventions on regional brain areas

To determine the effects of interventions on brain areas associated with the physiopathological process of AD, we selected only the results including a sham procedure and for participants with CI (Supplementary Figure 1). We chose to focus on participants with CI as we assumed that the effect of interventions might be different between participants with nCI or CI. We anticipated that the effect of interventions on participants with CI would be more homogeneous since they all must have, at least, objective cognitive impairment.

## 3. Results

### 3.1. Flowchart and characteristics of the population

**Figure 1:**
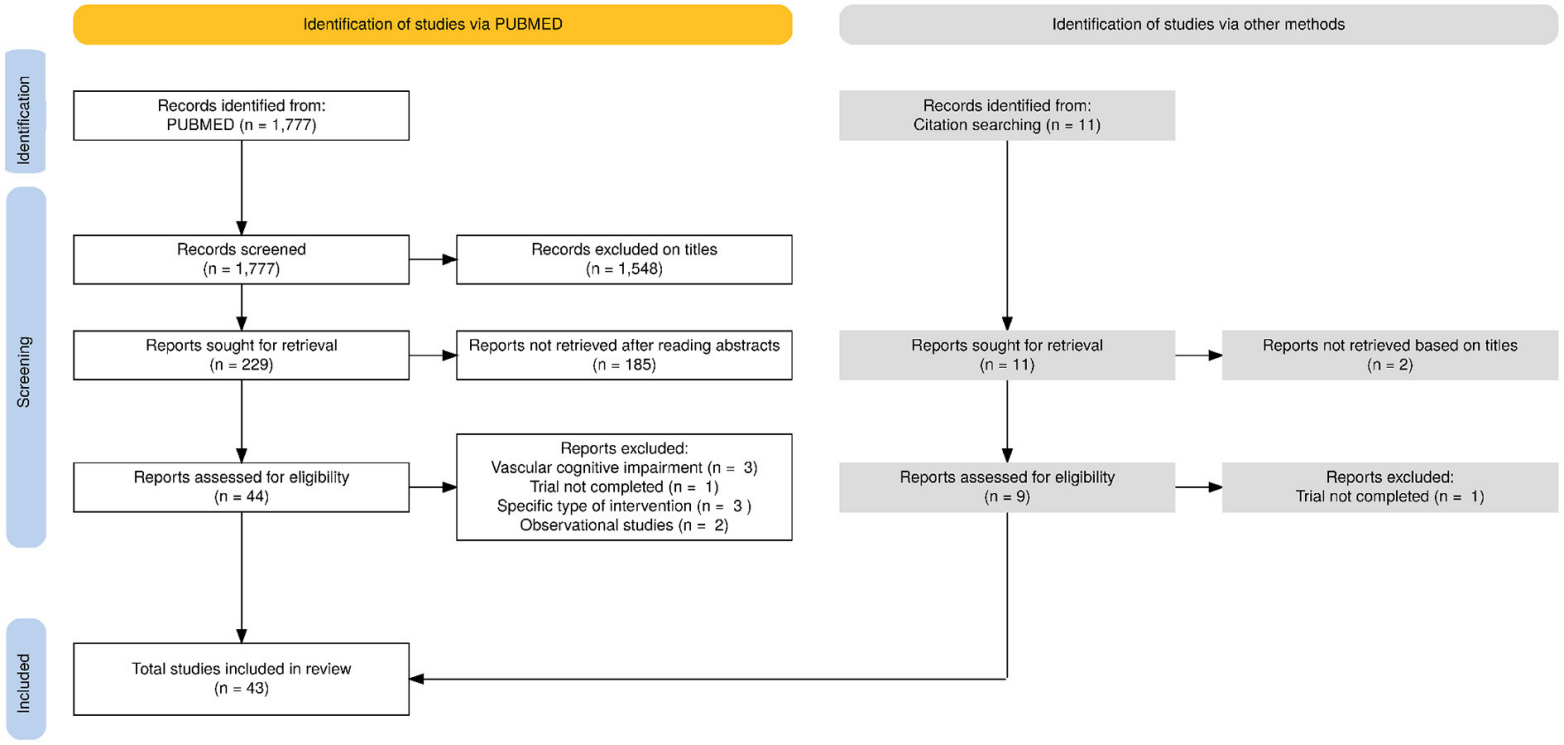
PRISMA flowchart for the selection of the articles evaluated in this review.

From the 1,788 identified articles, 43 (PE, *n* = 11; nutrition/diet, *n* = 10; CT, *n* = 13 and MD intervention, *n* = 9) met our inclusion criteria (Figure 1). Articles that were not selected after full-text review are described in the Supplementary Table 2. Thirty-three publications included participants with CI, nine, participants with nCI, two, both types and one, a mix of both cognitive status.

The populations with nCI exhibited one of the following risk factors: (1) subjective memory complaints/impairment (SMC/SMI) (*n* = 3; Youn et al., 2009; Risacher et al., 2015; Na et al., 2018); (2) “probable MCI” defined by a mini-mental state examination (MMSE) score within normal range but a reduced Montreal cognitive assessment (MoCA) score (*n* = 1; ten Brinke et al., 2015); (3) at-risk for dementia based on the CAIDE score (Cardiovascular Risk Factors, Aging, and Incidence of Dementia; Kivipelto et al., 2006) with cognitive performance at average or slightly lower level than expected according to Finnish population norms for Consortium to Establish a Registry for Alzheimer's Disease (CERAD) neuropsychological battery (Moms et al., 1989) (*n* = 2; Stephen et al., 2019, 2020); (4) limitations in one instrumental activity of daily living or slow gait speed or spontaneous memory complaints (*n* = 1; Delrieu et al., 2020); (5) high systolic blood pressure (*n* = 1; van Dalen et al., 2017); (6) elevated amyloid load or subthreshold amyloid levels [cerebral-to-cerebellar standard uptake value ratio (SUVR) threshold > 1.0; *n* = 1; Vidoni et al., 2021], or (7) hypertension and elevated amyloid load or subthreshold amyloid levels (SUVR > 1.0, see Vidoni et al., 2021) (*n* = 1; Kaufman et al., 2021).

Studies with CI participants included populations with risk factors for AD, that is (1) amnestic or non-amnestic MCI participants (Petersen et al., 1999) and (2) participants with objective CI associated with neurodegeneration biomarkers based on imaging [medial temporal lobe atrophy or hypometabolism on 18F-fluorodeoxyglucose (18F-FDG) positron emission tomography (PET) data] or CSF compounds (abnormal levels of Aβ peptides, Aβ_1 − 42_, Aβ_1 − 40_, Aβ_1 − 42/1 − 40_ ratio; t-Tau, or p-Tau_181_; *n* = 3; Dubois et al., 2007; Albert et al., 2011).

Multiple imaging modalities were used to assess the effect of interventions with brain gray matter (GM) structure being the most commonly evaluated (58%, Table 3). Neuroimaging biomarkers of GM, concerning either the whole brain, regional volume or atrophy, were obtained from T1-weighted MRI images. Different types of imaging biomarkers associated with white matter (WM) structure were used. Volumes of white matter hyperintensities (WMH) were obtained from fluid attenuated inversion recovery (FLAIR) sequences; metrics of WM tracts' integrity, such as fractional anisotropy (FA), were acquired from diffusion tensor imaging (DTI) analyses. Brain perfusion was evaluated using arterial spin labeling (ASL) and dynamic susceptibility contrast (DSC) perfusion imaging. One study used ultrasound imaging. Brain function was evaluated using task-based functional magnetic resonance imaging (t-fMRI) and resting state functional magnetic resonance imaging (rs-fMRI). Brain amyloid load was evaluated using 18F-AV-45 (florbetapir) PET imaging. PET imaging was also used to assess glucose metabolism. Eventually, one study used proton nuclear magnetic resonance (NMR) spectroscopy to analyze the impact of intervention on other metabolites: choline compounds, gamma-aminobutyric acid (GABA), glutamate-glutamine (Glx), and N-acetyl aspartate and N-acetylaspartyl-glutamate (NAA-NAAG).

**Table 3:** Among the 43 included studies, number of studies evaluating an imaging modality.

	**GM sMRI**	**WM sMRI^a^**	**fMRI^b^**	**Amyloid PET imaging**	**Perfusion MRI^c^**	**Ultrasound^d^**	**Glucose met. PET imaging**	**Other met. imaging^e^**
PE	4	2	3	1	3	1	1	0
Nutrition	8	1	1	0	2	0	2	1
CT	7	3	6	0	0	0	3	1
MD	6	3	2	0	1	0	1	0
Total	25	9	12	1	6	1	7	2

^a^Evaluation of white matter integrity with diffusion weighted imaging or evaluation of white matter hyperintensities on T2-FLAIR MRI.

^b^Task- or resting state-fMRI.

^c^ASL or DSC-MRI.

^d^Doppler ultrasound.

^e^Either PET or proton magnetic resonance spectroscopy.

ASL, arterial spin labeling; CT, cognitive training; DSC, dynamic susceptibility contrast; FLAIR, fluid attenuated inversion recovery; fMRI, functional MRI; GM, gray matter; MD, multidomain; Met, metabolism; MRI, magnetic resonance imaging; PE, physical exercise; PET, positron emission tomography; WM, white matter.

According to the notation criteria, 88% of the studies ranged from good to high quality (*n* = 38), only a few were considered of “limited” quality (*n* = 5; Table 2). Criteria #7 and #8, respectively corresponding to the intention-to-treat analysis and the description of drop-outs, were the least fulfilled. Around 60% of the studies were not sufficiently powered. Concerning imaging criteria, only one third of the studies specified that a control quality procedure was applied and only 46% of the studies applied multiple correction.

### 3.2. Types of interventions and effect on brain imaging biomarkers

#### 3.2.1. Physical exercise

From the 11 studies evaluating PE (Table 4), four were of high quality and seven of good quality (Table 2). A majority examined aerobic training and only one considered resistance training (ten Brinke et al., 2015). PE extended over 12–52 weeks, frequency ranged from 2 to 5 times a week and session length varied from 25–30 to 60 min, which approximately corresponds to the recommendations of the WHO (World Health Organization, 2019). Mean participants' age ranged from 65 to 81 years. The proportion of females varied from 39 to 83%, with one study including exclusively women (ten Brinke et al., 2015). Three publications originated from the Aerobic Exercise Training in Mild Cognitive Impairment (AETMCI) study (Tarumi et al., 2020; Thomas et al., 2020; Tomoto et al., 2021), two from the Alzheimer's Prevention through Exercise study (APEx) (Kaufman et al., 2021; Vidoni et al., 2021) and three originated from the same sample (Smith et al., 2013; Chirles et al., 2017; Alfini et al., 2019).

**Table 4:** Physical exercise (PE) interventions.

**References**	**Population(s)**	**Mean age (sd) Gender (F)**	**Intervention description**	**Intervention length/freq**.	**Imaging data at follow up**	**Main findings**	**Note**
Vidoni et al. (2021)	CN, elevated cerebral amyloid or SUVR > 1.0 *N* = 117	I: 71.2 ± 4.8 70.5% (F) C: 72.2 ± 5.3 61.5% (F)	I: AE (treadmill walking) C: standard exercise public health information	52 wk 150' over 3–5 session/wk	52 wk Amyloid im.: *N* = 109 GM sMRI: *N* = 104	• I ↑ cardiorespiratory fitness. • No effect of I on executive function, verbal memory, visuospatial function. • No effect of I on global cerebral amyloid load, and whole brain or hipp. vol.	14.0 (93%)
ten Brinke et al. (2015)	Probable MCI *N* = 39	I1: 76.07 ± 3.43 100% (F) I2: 73.75 ± 3.72 100% (F) C: 75.46 ± 3.93 100% (F)	I1: AE I2: Resistance training C: Balance and tone training	6 mo 60' session 2x/wk	6 mo GM sMRI: *N* = 39	• I1 ↑ total hipp. vol. •No effect of I2 on GM sMRI outcomes. • Increase in left hipp. vol. associated with poorer performance in the number of words recalled post-interference (RAVLT).	14.0 (88%)
Tarumi et al. (2020)	aMCI *N* = 36	I: 67 ± 7 44 % (F) C: 66 ± 7 50% (F)	I: AE C: stretching and toning	12 mo 25–30' session 3x/wk to 30–40' session 4–5x/wk	12 mo WMH im. and DTI: *N* = 36	• I minimally improves cognition (letter fluency performance). • No effect of I on WM lesions or metrics derived from DTI. • Peak oxygen uptake ↑ associated with attenuated ↑ in MD and AxD. • No correlation between cognitive test scores and peak oxygen uptake or WM integrity.	14.0 (88%)
Tomoto et al. (2021)	aMCI *N* = 52	I: 64.8 ± 6.4 55% (F) C: 66.1 ± 6.8 53% (F)	See Tarumi et al. (2020)	See Tarumi et al. (2020)	12 mo Duplex ultra- sonography and transcranial doppler and GM sMRI and WMH im*: *N* = 35	• I minimally improves cognition (letter fluency performance). • I ↓ carotid β-stiffness index and CBF pulsatility and ↑ global CBF. • ↑ of cardiorespiratory fitness associated with ↑ of CBF, and ↓ of carotid β-stiffness and CBF pulsatility. • No effect of I on WMH (see Tarumi et al., 2020) and total brain or hipp. vol. but global ↑ of WMH vol. and ↓ of hipp. and whole brain vol.	12.0 (80%)
Yogev-Seligmann et al. (2021)	aMCI *N* = 27	I: 70.84 ± 5.53 38.5% (F) C: 71.92 ± 6.4 50% (F)	I: AE C: balance, gross motor coordination and light toning exercise	16 wk 40' session 3x/wk	16 wk t-fMRI: *N* = 27	• I ↑ front. activity during memory encoding (vs. C). • I ↑ neural synchronization in front., ins., cing., par., occ. and temp. cortices. I ↓ activity during memory encoding for C in front., par., temp., occ. and cer. areas. • No effect of I on cognition but ↑ of cardiorespiratory fitness associated with ↑ brain activity in front. areas for I.	12.0 (75%)
Kaufman et al. (2021)	CN, elevated cerebral amyloid or SUVR > 1.0 and hypertensive *N* = 44	71.8 ± 5.4 61% (F)	See Vidoni et al. (2021)	See Vidoni et al. (2021)	52 wk Perf. MRI: *N* = 44	• I ↑ HBF in APOEϵ4 carriers. • For APOEϵ4 carriers, ↓ SBP associated with ↑ HBF. • After I, correlation between HBF and verbal memory for APOEϵ4 carriers; no interaction between I and APOEϵ4 status on visuospatial functioning, executive functioning or verbal memory.	11.0 (73%)
Thomas et al. (2020)	aMCI *N* = 30	I: 66.4 ± 6.6 46.7% (F) C: 66.1 ± 7.2 46.7% (F)	See Tarumi et al. (2020)	See Tarumi et al. (2020)	12 mo Perf. MRI and GM sMRI: *N* = 30	• LM and cardiorespiratory fitness improves after I group. • No effect of I on GMV. • I ↑ CBF in ACC, ↓ CBF in PCC. • For all participants, CBF in ACC and front. areas correlated with LM.	11.0 (69%)
Alfini et al. (2019)	MCI *N* = 15 HOA *N* = 17	MCI: 80.5 ± 5.8 60.0% (F) HOA: 76.5 ± 7.2 82.4% (F)	I: AE	12 wk 30' session 4x/wk	12 wk Perf. MRI: *N* = 32	• MCI and HOA both improve on RAVLT and COWAT tests after I. • Elevated CBF at baseline in left ins. for MCI vs. HOA attenuated after I. • For MCI, negative correlation between COWAT and CBF in the left ins. and ACC. • For MCI, I ↓ CBF in left ACC and right inf. front. gyrus. For HOA, I ↑ CBF in right ACC.	11.0 (69%)
Chirles et al. (2017)	MCI *N* = 16 HOA *N* = 16	MCI: 79.6 ± 6.8 63% (F) HOA: 76.1 ± 7.2 81% (F)	See Alfini et al. (2019)	See Alfini et al. (2019)	12 wk rs-fMRI: *N* = 32	• MCI and HOA both improve on verbal memory (RAVLT) after I. • I ↑ connectivity between PCC/PCu and left postcentral gyrus for MCI and HC; ↑ connectivity between PCC/PCu and right par. lobe for MCI and ↓ connectivity for HOA.	10.0 (62%)
Smith et al. (2013)	MCI *N* = 17 HOA *N* = 18	MCI: 78.7 ± 7.5 59% (F) HOA: 76.0 ± 7.3 83% (F)	See Alfini et al. (2019)	See Alfini et al. (2019)	12 wk t-fMRI: *N* = 34	• MCI and HOA improve on RAVLT Trial 1, and peak aerobic capacity after I. • For MCI and HC, I ↓ task activation for left precentral gyrus, left lentiform, left PCu, right sup. par. lobule/angular gyrus, right sup./mid. temp. gyrus, left lat. occ. gyrus and left cer.	10.0 (62%)
Henrique de Gobbi Porto et al. (2015)	MCI *N* = 40	70.3 ± 5.4 77.5% (F)	I: AE	24 wk 30–50' session 2x/wk	24 wk Glc met. im.: *N* = 40	• I improves cognition (ADAS-COG). • I ↓ glc metabolism in ACC which correlates with improvement of visuospatial function/attentional functions (cROCF). • I ↓ glc metabolism in ACC which negatively correlates with increase in the right retrosplenial cortex.	8.0 (50%)

↓, decrease; ↑, increase; ACC, anterior cingulate cortex; AD, Alzheimer's disease; ADAS-COG, Alzheimer's disease assessment scale-cognitive subscale; APOE, apolipoprotein E; AE, aerobic exercise; aMCI, amnestic mild cognitive impairment; AxD, axial diffusivity; C, control condition; CBF, cerebral blood flow; Cer, cerebellum; Cing, cingulate; cROCF, copy of the Rey-Osterrieth Complex Figure; CN, cognitively normal; COWAT, controlled oral word association test; F, female; Front, frontal; Glc, glucose; GM sMRI, gray matter structural magnetic resonance imaging; GMV, gray matter volume; Hipp, hippocampus; HBF, hippocampal blood flow; HOA, healthy older adults; I, intervention; Inf, inferior; Ins, insula; Im, imaging; Lat, lateral; LM, logical memory; M, male; MCI, mild cognitive impairment; Met, metabolism; MD, mean diffusivity; Mid, middle; Mo, months; MRI, magnetic resonance imaging; Occ, occipital; Par, parietal; PCC, posterior cingulate cortex; PCu, precuneus; Perf, perfusion imaging; RAVLT, rey auditory verbal learning test; rs-fMRI, resting state functional magnetic resonance imaging; Sup, superior; SUVR, standard uptake value ratio; Temp, temporal; t-fMRI, task functional magnetic resonance imaging; Vol, volume; Wk, week; WM, white matter; WMH, white matter hyperintensities.

*In order to simplify, we reported the minimum number of data provided for the different imaging metrics.

##### 3.2.1.1. Effect of PE on brain structure and amyloid load

For participants with nCI, the majority of the studies on PE reported null results (two studies, k = 4, all with sham) on brain structure (k = 3) or amyloid imaging (k = 1; Table 5). The only positive study revealed an increased hippocampal volume (HV) in participants with “probable” MCI after 6 months of PE (ten Brinke et al., 2015). This increase in HV was counterintuitively associated with reduced verbal memory and learning performance (ten Brinke et al., 2015). However, 6 months of resistance training (~weight lifting) did not impact HV in those participants (ten Brinke et al., 2015). A longer duration (52 weeks) of aerobic exercise did not modify HV, brain volume nor global cerebral amyloid load in older adults with elevated amyloid load (Vidoni et al., 2021).

**Table 5:** Positive and null results for each intervention (see Figure 2) and according to neuroimaging modalities.

	**+(C)**	**-(C)**	**+(NC)**	**-(NC)**
**PE**				
nCI (*N* = 2, k = 4)	GM sMRI (k = 1, q = 88%)	GM sMRI (k = 2, q = 90%) Amyloid im. (k = 1, q = 93%)	k = 0	k = 0
CI (*N* = 8, k = 11)	Ultrasound (k = 1, q = 80%) fMRI (k = 1, q = 75%) Perf. MRI (k = 1, q = 69%)	WM sMRI (k = 2, q = 88%) GM sMRI (k = 2, q = 74%)	fMRI (k = 2, q = 62%) Perf. MRI (k = 1, q = 69%) Glc met. im. (k = 1, q = 50%)	k = 0
**Nutrition**				
nCI (*N* = 1, k = 2)	k = 0	Perf. MRI (k = 1, q = 62%) GM sMRI (k = 1, q = 62%)	k = 0	k = 0
CI (*N* = 7, k = 10)	GM sMRI (k = 3, q = 83%) Perf. MRI (k = 2, q = 56%) fMRI (k = 1, q = 69%) Glc met. im. (k = 1, q = 20%)	GM sMRI (k = 2, q = 66%) WM sMRI (k = 1, q = 69%)	k = 0	k = 0
**CT**				
nCI (*N* = 2, k = 5)	WM sMRI (k = 1, q = 62%)	GM sMRI (k = 1, q = 62%)	k = 0	Glc met. im. (k = 1, q = 50%) GM sMRI (k = 1, q = 50%) WM sMRI (k = 1, q = 50%)
CI (*N* = 11, k = 21)	GM sMRI (k = 3, q = 46%) fMRI (k = 5, q = 59%) Glc met. im. (k = 1, q = 1, q = 62%) Other met. im. (k = 1, q = 50%)	Other met. im. (k = 3, q = 50%) Glucose met. (k = 1, q = 56%)	GM sMRI (k = 3, q = 50%) WM sMRI (k = 1, q = 50%) fMRI (k = 2, q = 53%) Glc met. im. (k = 1, q = 50%)	k = 0
**MD**				
nCI (*N* = 4, k = 5)	WM sMRI (k = 1, q = 69%)	WM sMRI (k = 2, q = 83%) Glc met. im. (k = 1, q = 81%) GM sMRI (k = 1, q = 81%)	k = 0	k = 0
CI (*N* = 4, k = 6)	GM sMRI (k = 2, q = 66%) Perf. MRI (k = 1, q = 88%) fMRI (k = 1, q = 88%)	GM sMRI (k = 1, q = 88%)	GM sMRI (k = 1, q = 23%)	k = 0

Results on WM can include results on white matter integrity evaluated using diffusion weighted imaging or results on WM hyperintensity. These two types of results are considered and counted separately but grouped under the same appellation of WM structure in this table. For a set of k results, the mean quality q of the studies from which the results were reported was specified.

Were not included in this table results from intervention intermediate timepoints (Delrieu et al., 2020: results at 6 months, Soininen et al., 2017) or followup measures (Broadhouse et al., 2020: results at 18 months), from a mixed sample of participants (Neth et al., 2020: ketone uptake and glucose metabolism), and studies reporting only results from correlation analyses (Vermeij et al., 2016; Anderson-Hanley et al., 2018; Hama et al., 2020), or analyzing specific subgroups within a clinical trial (Jernerén et al., 2015; Kaufman et al., 2021).

+ (C), number of positive results from studies including a control group; -(C), number of null results from studies including a control group; + (NC), number of positive results from studies without a control group; - (NC), number of null results from studies without a control group; CI, cognitive impairment; CT, cognitive training; fMRI, functional MRI; GM, gray matter; Im, imaging; k, number of results from all modalities of *N* studies; MD, multidomain; Met, metabolism; MRI, magnetic resonance imaging; *N* = number of studies; nCI, no cognitive impairment; PE, physical exercise; q, mean quality score; sMRI, structural MRI; WM, white matter.

**Figure 2:**
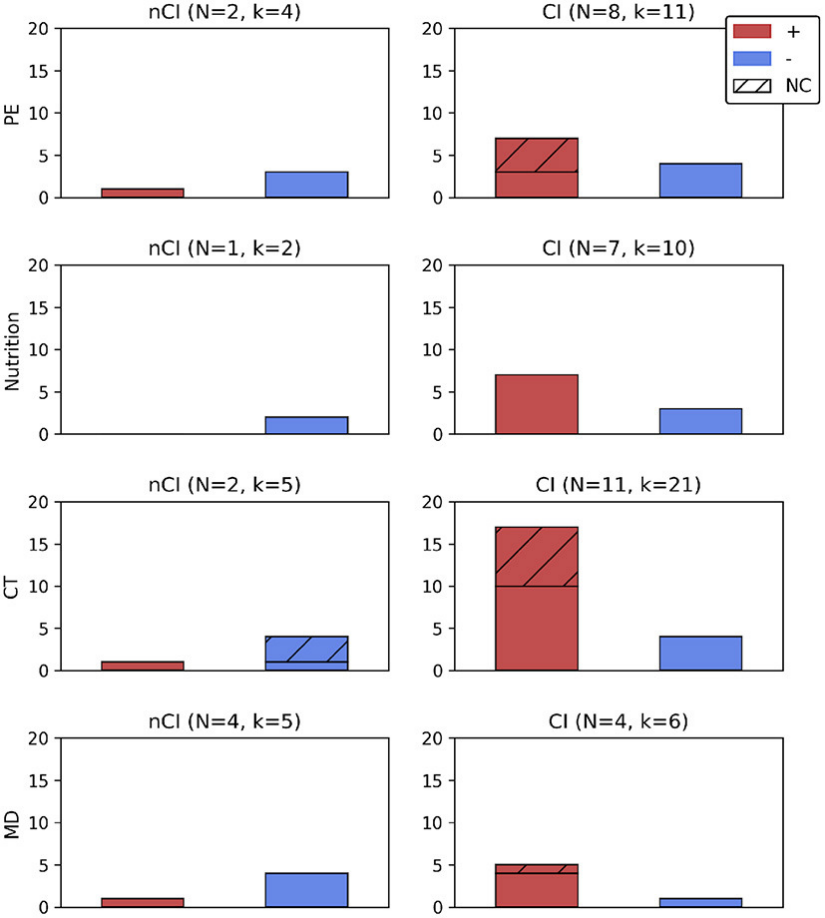
Number of positive or null results according to the type of intervention, with or without a sham intervention, and presented according to the cognitive profile (nCI, CI). For *N* studies administering an intervention all results from all imaging modalities (k) were reported. Were not included in this figure results from intervention intermediate timepoints (Delrieu et al., 2020: results at 6 months, Soininen et al., 2017) or followup measures (Broadhouse et al., 2020: results at 18 months), from a mixed sample of participants (Neth et al., 2020: ketone uptake and glucose metabolism), and studies reporting only results from correlation analyses (Vermeij et al., 2016; Anderson-Hanley et al., 2018; Hama et al., 2020), or analyzing specific subgroups within a clinical trial (Jernerén et al., 2015; Kaufman et al., 2021). +, number of positive results (effect of an intervention); −, number of null results (lack of effect from an intervention); CI, cognitive impairment; CT, cognitive training; k, number of results (all imaging modalities); MD, multidomain; *N*, number of studies; NC, results from studies without a sham group; nCI, no cognitive impairment; PE, physical exercise.

For participants with CI, and all neuroimaging modalities being taken into account, positive results outweigh null results. However, when considering only studies with a sham procedure, the number of positive and null results was almost even (3 vs. 4; Figure 2). For publications evaluating brain morphology, all had a sham intervention and none reported an effect of PE [-(C), Table 5]. Twelve months of aerobic exercise did not modify WMH (Tarumi et al., 2020; Tomoto et al., 2021) nor total brain volume (Tomoto et al., 2021) or HV (Thomas et al., 2020; Tomoto et al., 2021) and other brain regions volumes (Thomas et al., 2020) for amnestic MCI (aMCI) participants. WMH are of vascular origin and an indication of demyelination and axonal damage (Wardlaw et al., 2015) and highly associated with preclinical AD (Kandel et al., 2016). WM integrity, as evaluated by DTI, was equally not affected (Tarumi et al., 2020).

##### 3.2.1.2. Effect of PE on brain function, perfusion, and metabolism

For participants with nCI, only one study reported that PE modified cerebral blood flow (CBF) and specifically for a subgroup of APOE ϵ4 participants (Kaufman et al., 2021).

For participants with CI, either MCI or aMCI, PE of various duration modified brain activity, glucose metabolism, and CBF (Table 5).

Studies involving patients with MCI reported positive effects of PE on brain activity, glucose metabolism and CBF but did not compare this effect to a sham intervention [Table 5, + (NC)]. Twelve weeks of PE increased functional connectivity (FC) of the posterior cingulate cortex (PCC)/Precuneus (Chirles et al., 2017). The PCC/Precuneus is a central node of the default mode network (DMN), a functional brain network associated with internally focused tasks such as autobiographical memory retrieval (Buckner et al., 2008) and which dysfunction has been observed in early AD patients (Simic et al., 2014). This increase of FC of the PCC/Precuneus might be linked to mechanisms of neural compensation that appear with age-related or pathological brain networks' impairment (Chirles et al., 2017). For patients of the same cohort, task activation was decreased in multiple brain regions during a semantic memory retrieval task, implying that neural efficiency was improved and suggesting a better recruitment of neural resources after PE (Smith et al., 2013). Participants of the aforementioned cohort (Smith et al., 2013) exhibited decreased exercise-induced CBF at baseline in the left insula—a brain region involved in higher order cognitive processes and affected early by AD—until it reached healthy controls levels (Alfini et al., 2019). PE additionally decreased CBF in the left anterior cingulate cortex (ACC) and in inferior frontal regions (Alfini et al., 2019). Decrease of CBF has been associated with decreased cognitive performance in aging (Leeuwis et al., 2018) and commonly observed in AD patients; it has been hypothesized that PE could alleviate this decrease (Tarumi and Zhang, 2018). The decrease in CBF observed by Alfini et al. (2019) thus seems surprising. However, hyperperfusion has also been observed in older adults with increased risk of AD, and intervention may help restore a declining neurovascular system that initially showed abnormally high CBF.

PE intervention decreased glucose metabolism in the ACC after 24 weeks of training (Henrique de Gobbi Porto et al., 2015). This decrease appeared to reflect better brain functioning as it was associated with an improvement of the visuospatial and attentional function. Moreover, it was related to an increase of glucose metabolism in the retrosplenial cortex, which is part of the DMN, suggesting opposite mechanisms in the two areas (Henrique de Gobbi Porto et al., 2015).

All studies with aMCI patients included a sham condition and showed that PE affected both CBF and brain function. CBF was globally increased after 12 months of aerobic training (Tomoto et al., 2021) and regionally modulated with an increase in the ACC and a decrease in the PCC, suggesting a posterior-to-anterior shift of brain perfusion (Thomas et al., 2020). Eventually, activation in frontal areas was increased for aMCI following a memory encoding task and after 16 weeks of PE, possibly reflecting a compensatory mechanism (Yogev-Seligmann et al., 2021).

#### 3.2.2. Nutrition/diet

From the 10 studies on nutrition (Table 6), two of them were considered of limited quality, five of good quality and three of high quality (Table 2). Interventions comprised B vitamins (*n* = 2), folate (*n* = 1), resveratrol (*n* = 1), medical food Souvenaid (*n* = 3), omega-3 (*n* = 1), algal docosahexaenoic acid (DHA) (*n* = 1) supplementations, and modified Mediterranean-ketogenic (MMKD) and American Heart Association diets (AHAD) (*n* = 1).

**Table 6:** Nutritional interventions.

**References**	**Population(s)**	**Mean age (sd) Gender (F)**	**Intervention description**	**Intervention length/freq**.	**Imaging data at follow up**	**Main findings**	**Note**
Soininen et al. (2017)	prAD *N* = 311	I: 71.3 ± 7.0 47% (F) C: 70.7 ± 6.2 54% (F)	I: LipiDiDiet (medical food Souvenaid) C: Control drink	24 mo 125 mL daily supplementation	24 mo GM sMRI: *N* = 200	• No effect of I on NTB composite z-score, but less worsening on CDR-SB. • I reduced hipp. atrophy and ventricles enlargement; no effect on whole brain volume.	14.0 (93%)
Soininen et al. (2021)	prAD *N* = 311	I : 71.3 ± 7.0 47% (F) C : 70.7 ± 6.2 54% (F)	I: LipiDiDiet (medical food Souvenaid) C: Control drink	36 mo See Soininen et al. (2017)	36 mo GM sMRI *N* = 75	• I reduced decline in CDR-SB, NTB composite z-score and NTB memory domain. • Less deterioration of hipp., whole brain and less ↑ of ventricular volumes after I.	14.0 (93%)
Smith et al. (2010)	MCI *N* = 168	I: 77.0 ± 5.2 58.8% (F) C: 76.2 ± 4.5 62.7% (F)	I: B vitamin C: Placebo	2 years daily supplementation of folic acid (0.8 mg), vitamin B-6 (20 mg), vitamin B-12 (0.5 mg)	2 years GM sMRI: *N* = 168	• I ↓ brain atrophy. • I had greater effect with higher baseline HCy levels. • Negative association between atrophy rate and cognition.	13.0 (87%)
Zhang et al. (2016)	MCI *N* = 240	I: 74.49 ± 2.65 64.17 % (F) C: 74.57 ± 3.31 65.83% (F)	I: Algal DHA C: Corn oil	12 mo daily supplementation of 2g of ω3 intake (algal DHA)	12 mo GM sMRI: *N* = 240	• I ↑ cognitive capacity (Full-Scale IQ, WAIS-RC Information and Digit Span tests). • I ↑ hipp. and total cerebrum volumes; no effect on volume. • Cognition correlated with total hipp. and ventricles volumes.	11.0 (69%)
Köbe et al. (2017)	aMCI *N* = 40	I : 65 ± 9 56% (F) C : 69 ± 7 50% (F)	I: Resveratrol C: Olive oil	26 wk daily supplementation, 200 mg resveratrol and 350 mg quercetin	26 wk GM sMRI & DTI & rs-fMRI: N=30	• No effect on memory. • I ↑ FC between hipp. and angular cortex. • No effect on hipp. MD • Preserves hipp. volume (trend).	11.0 (69%)
Jernerén et al. (2015)	MCI *N* = 168	76.6 (75.9, 77.3) (95% CI) 60.7% (F)	See Smith et al. (2010)	See Smith et al. (2010)	2 years GM sMRI: *N* = 168	• I affects conjointly with baseline EPA and DHA status atrophy. • I more efficient in subjects with high baseline EPA and DHA status; no effect on subjects with low ω3.	9.0 (64%)
Neth et al. (2020)	MCI *N* = 9 SMC *N* = 11	SMC: 64.9 ± 7.9 82 % (F) MCI: 63.4 ± 4.0 67 % (F)	I: Mediterranean ketogenic diet (MMKD) C: American Heart Association Diet (AHAD)	first diet (6wk) then washout (6wk) then second diet (6 wk). Target macronutrient composition: 5–10% carbohydrate, 60–65% fat, 30% protein	18 wk Perf. MRI & GM sMRI: *N* = 20 Glc& Ketone body met. im.: *N* = 5	• MCI and SMC improved on FCSRT after I and C, but not on ADAS-Cog story recall. • I ↑ and CSF Aβ42/tau ratio levels for MCI and SMC and ↓ tau levels for MCI. • No effect of I or C on GMV for MCI nor for SMC. • No effect of I or C on glc met, but ↑ of ketone body uptake after I (pooled MCI and SMC). • I ↑ perfusion in left PH and right temp. areas (due to MCI).	10.0 (62%)
Hama et al. (2020)	MCI with folate deficiency *N* = 45	79.7 ± 7.9 37.8% (F)	I : Folate supplementation	28–63 days 5 mg daily supplementation	Followup (28–63 days): GM sMRI: *N* = 30	• I ↓ HCy levels. • I ↑ MMSE score. • Baseline hipp. atrophy not associated with change in MMSE.	4.0 (31%)
Manzano Palomo et al. (2019)	MCI *N* = 41	I: 72.18 ± 6.34 47.1% (F) C: 68.08 ± 8.54 66.7% (F)	I: medical food Souvenaid C: no treatment	1 year n.s	1 year Glc met. im.: N = 39	• I preserved memory performance, executive function and attention. No effect of I on rate of progression of dementia, but stabilized/improved evolution reported in SCS. • No effect on vascular risk factors but hypercholesterolemia ↓ after I. • Worsening of glc met. in C only.	3.0 (20%)
Schwarz et al. (2017)	aMCI *N* = 13	I: 67 ± 9 38% (F) C: 66 ± 9 60% (F)	I: ω3 diet C: Sunflower oil	26 wk 2,200 mg daily intake	26 wk Perf. MRI: *N* = 13	• I ↑ CBF and volume in post. cortical regions.	7.0 (50%)

↓, decrease; ↑, increase; Aβ, amyloid β; ω3, Omega-3; AD, Alzheimer's disease; ADAS-Cog, Alzheimer's disease assessment scale-cognitive subscale; aMCI, amnestic mild cognitive impairment; C, control condition; CBF, cerebral blood flow; CDR-SB, clinical dementia rating-sum of boxes; CSF, cerebrospinal fluid; DHA, docosahexaenoic acid; DTI, diffusion tensor imaging; EPA, eicosapentaenoic acid; F, female; FC, functional connectivity; FCSRT, free and cued selective reminding test; glc, glucose; GMV, gray matter volume; GM sMRI, gray matter structural magnetic resonance imaging; HCy, homocysteine; hipp, hippocampus; I, intervention; Im, imaging; Iq, intelligence quotient; M, male; MCI, mild cognitive impairment; MD, mean diffusivity; Met, metabolism; MMSE, mini-mental state examination; Mo, months; MRI, magnetic resonance imaging; NTB, neuropsychological test battery; n.s, not specified; Perf, perfusion imaging; PH, parahippocampal; Post, posterior; prAD, prodromal AD; rs-fMRI, resting state functional magnetic resonance imaging; SCS, subjective changing scale; SMC, subjective memory complaint; temp, temporal; WAIS-RC, wechsler adult intelligence scale-revised; Wk, week.

B vitamins could mitigate the increase in homocysteine plasma levels which have been associated with brain atrophy, among other deleterious processes (Kennedy, 2016). Resveratrol has been shown to increase brain neurogenesis (Gomes et al., 2018), decrease amyloid deposition (Ashrafizadeh et al., 2020), and reduce tau hyperphosphorylation (Yan et al., 2020) and appears as a promising means of prevention (Tosatti et al., 2022). Souvenaid is a medical food which includes a mix of nutrients: DHA, eicosapentaenoic acid (EPA), uridine monophosphate, choline, B12, B6, C, E vitamins, folic acid, phospholipids and selenium, and which has been shown to improve memory function (van Wijk et al., 2013). Omega-3 intake, including either DHA or EPA, have been shown to be beneficial at the onset of AD (Canhada et al., 2018) and to improve cognition in older adults (Swanson et al., 2012). Ketogenic diets have been shown to improve cognition in patients with AD, possibly by reducing amyloid burden (Rusek et al., 2019).

Both the studies from Smith et al. (2010) and Jernerén et al. (2015) originated from the VITACOG trial testing the impact of a B vitamins intervention on AD. The two publications by Soininen et al. (2017, 2021) came from the LipiDiDiet trial investigating the effect of a 24–36 month intervention with Souvenaid. Intervention duration varied from 28 days to 36 months and most studies used daily supplementation. Participants' mean age ranged from 63 to 80 years, and the proportion of females varied from 38 to 82%.

##### 3.2.2.1. Description of the effect of nutrition on brain structure

The effects of nutritional interventions were all assessed in trials including a sham intervention (Figure 2). Only one study examined the effect of nutrition on the brain structure of participants with nCI (Neth et al., 2020; Table 5). This study reported that 6 weeks of MMKD did not impact gray matter volume (GMV) for subjects with SMC (Neth et al., 2020).

For patients with CI, and regardless of the type of neuroimaging biomarker, the seven studies examining the impact of nutritional interventions obtained more positive than null results (7 vs. 3; Figure 2). The results related to structural analyses were mixed. They were positive after B vitamins, DHA, and souvenaid intake but not after MMKD or resveratrol intake (Table 5). The two publications obtained from the VITACOG trial reported the impact of 2 years of B vitamins intervention on MCI patients. Those patients exhibited reduced brain atrophy while the responsiveness to B vitamins was increased in participants with the highest baseline homocysteine levels (Smith et al., 2010) and ω-3 fatty acid status (Jernerén et al., 2015). MCI participants were also positively receptive to algal DHA intervention which increased total brain volume and HV after 12 months of daily supplementation (Zhang et al., 2016). However, 6 weeks of MMKD did not modify the GMV of MCI patients (Neth et al., 2020). For MCI with folate deficiency (< 3.6 ng/mL), 28–63 days of folate supplementation improved cognition (MMSE). Nevertheless, no relation could be established between this improvement and participants' baseline atrophy (Hama et al., 2020).

For patients with aMCI, 26 weeks of resveratrol intervention did not affect HV, though a statistical trend was observed (*p* = 0.06; Köbe et al., 2017). This intervention did not affect mean diffusivity in the hippocampus (Köbe et al., 2017). Eventually, for patients with CI together with positive biomarkers of neurodegeneration, two publications originating from the same trial and assessing the effect of 24–36 months of Souvenaid (Soininen et al., 2017, 2021) showed that it protected from hippocampal atrophy and ventricular enlargement. A decline in global brain atrophy was later detected at 36 months (Soininen et al., 2021).

##### 3.2.2.2. Description of the effect of nutrition on brain function, perfusion, and metabolism

The sole study including participants with nCI, a group mixing SMC with MCI, reported that 6 weeks of MMKD increased brain perfusion (Neth et al., 2020). When change in perfusion was analyzed according to the cognitive status, it revealed that this increase was driven by the MCI participants (Neth et al., 2020). MMKD intervention increased ketone body uptake while it did not modify glucose metabolism (Neth et al., 2020). Due to the limited size of the sample, the effect of the intervention on ketone body uptake and glucose metabolism could not be evaluated for each cognitive group individually.

Studies involving CI participants all reported an effect of the nutritional interventions on either brain metabolism, perfusion or function (Table 5). One year of Souvenaid preserved glucose metabolism in MCI, while hypometabolism was observed for the sham intervention (Manzano Palomo et al., 2019). Twenty-six weeks of ω-3 supplementation increased CBF in posterior regions for aMCI (Schwarz et al., 2017). Twenty-six weeks of resveratrol also affected aMCI participants by increasing FC between the hippocampus and the angular gyrus, two core areas of the DMN susceptible to be affected by neurodegenerative processes (Köbe et al., 2017).

#### 3.2.3. Cognitive training

Thirteen publications reported the impact of CT on neuroimaging biomarkers for at-risk for AD patients (Table 7). Eleven studies were considered of good quality while two were of limited quality (Table 2). The duration of CT varied from 3 days to 6 months, ranging from 1 to 5 sessions per week, and sessions' length ranged from 40 to 120 min. Participants' mean age ranged from 61 to 76 years, and the proportion of females varied from 20 to 90%. All studies originated from independent trials.

**Table 7:** Cognitive training (CT) interventions.

**References**	**Population(s)**	**Mean age (sd) Gender (F)**	**Intervention description**	**Intervention length/freq**.	**Imaging data at follow up**	**Main findings**	**Note**
Simon et al. (2018)	aMCI *N* = 30	I: 73.3 ± 5.9 73.3% (F) C: 71.0 ± 6.5 80% (F)	I: mnemonic strategy training C: education program	2 wk 1 h session 2x/wk	2 wk: t-fMRI (memory encoding task): *N* = 30	• Face-name memory improved post-training and at 1 and 3 mo after end of I; SUT recognition task improved at 1 mo and post training; SUT free recall at 1 mo of training. Self-reported improvement for I group in their memory abilities post-training. • I ↑ task-induced activation in the left ant. temp. lobe.	11.0 (69%)
Barban et al. (2017)	aMCI *N* = 23 Mild AD *N* = 22 HOA *N* = 25	aMCI I: 71.4 ± 6.6 30% (F) C: 72.8 ± 5.7 46% (F) Mild AD I: 76.4 ± 6 64.3% (F) C: 73.9 ± 4.7 63% (F) HOA I: 69.9 ± 5.6 66.7% (F) C: 71 ± 6.8 77% (F)	I: CCT (focused on memory, executive functions, attention, and reasoning) C: active control	3 mo of I followed by 3 mo of C or vice versa 1 h session 2x/wk	6 mo rs-fMRI: *N* = 61	• I improved memory on the whole sample (mainly driven by aMCI) and attention (mainly driven by mAD). • DMN FC : I ↑ connectivity of PCu for the whole sample, ↓ FC in med. sup. front. gyrus for aMCI (vs. HOA, FC ↑ for HOA), and ↓ FC in med. temp. lobe for mAD. • Whole brain FC : For aMCI, I ↑ BC of the orbito-front. region and ↓ BC of cer., and ↓ Th. ↔ hipp., Th. ↔ globus pallidus, and cer. ↔ cu FC. For mAD, ↑ BC of the right ant. cingu. and ↑ calcarine cortices ↔ left med. termp. lobe FC. No effect of I on FC for HOA.	11.0 (69%)
Li et al. (2019)	MCI *N* = 141	69.5 ± 7.3 39% (F) C : 71.5 ± 6.8 66.7% (F)	I: CCT (working and episodic memory speed of calculation, visual search, alertness, mental rotation, and images re-arrangement tasks) C: control group	6 mo 3–4 session/wk with a total of 120–160'	6 mo rs-fMRI: *N* = 141	• I improved cognition (MMSE, ACER Attention and Memory, CFT copy, CWST interference index) at 6 mo; no ≠ at 12 mo. • I ↑ regional activity in bilat. temp. poles, ins. and left PH areas at 6 mo.	11.0 (69%)
Belleville et al. (2011)	aMCI *N* = 15 HOA *N* = 15	aMCI: 70.13 ± 7.34 73.3% (F) HOA: 70 ± 7.26 66.7% (F)	I: episodic memory training	6 wk 2 h session 1x/wk	6 wk t-fMRI (verbal encoding and retrieval tasks): *N* = 30	• I improved word recall in MCI and HOA. • For MCI with encoding task, ↑ activation after I (par. lobule, front., cer., temp., ins. and basal ganglia areas). For retrieval task, ↑ activation (par., prefront., PCC, ins. and temp. areas). No effect on hipp target region. • For HOA, ↑ activation during retrieval and ↓ activation during encoding, after I. Effect on hipp target region. • I ↓≠ in activation between MCI and HOA in cing./med. front. gyrus and in the right par. lobe.	10.0 (62%)
Ciarmiello et al. (2015)	aMCI *N* = 30	I: 71.22 ± 7.66 60% (F) C: 71.95 ± 7.13 53.3% (F)	I: training on attention, executive functions, memory domains C: usual lifestyle with regular meetings with a psychologist	4 mo 45' session 2x/wk	4 mo Glc met. im.: *N* = 30	• No effect of I on cognition. • I modifies glc met. in front., temp. fusiform gyrus, caudate nuclei and ant. cing. areas. • For I, association between evolution of glc met. and cognitive performance, attention, and executive function.	10.0 (62%)
Youn et al. (2019)	SMC *N* = 201	I : 69.9 ± 5.10 64% (F) C: 69.11 ± 4.6 60% (F)	I: metamemory training C: general education on memory	10 wk 90' session 1x/wk	10 wk GM sMRI & DTI: *N* = 49	• After I, ↑ in long-term delayed free recall of verbal memory, categorical fluency, and in the Boston naming test. • I ↓ MD in left sup. longitudinal fasciculus, left corona radiata, left external capsule, corpus callosum, and left post. limb of the internal capsule. • No effect of I on FA, RD and AxD. • Trend for ↑ in prefront. cortex.	10.0 (62%)
Zhang et al. (2019)	aMCI *N* = 17	75.2 ± 3.8 64.7% (F)	I: CCT (reasoning, memory, visuospatial skill, language, calculation, and attention cognitive domains)	12 wk 1 h session 2x/wk	12 wk GM sMRI: *N* = 12	• No effect of I on cognition. • ↑ GMV in right angular gyrus. • Correlation between GMV in the right angular gyrus area and scores on the immediate recall component of the HVLT-R and on the BVMT-R.	8.0 (57%)
Park et al. (2019)	aMCI *N* = 50	I : 70.7 ± 7.5 52% (F) C : 69.7 ± 8.4 60% (F)	I: training mainly on memory, frontal lobe function, and orientation domains C: no cognitive training	12 wk Home-based cognitive intervention (daily 30' homework)+ hospital visit 1 day/wk	12 wk Glc met. im.: *N* = 32	• I improved performance on the COWAT test at 12 and 24 wk. • I had no effect on glc met.	9.0 (56%)
Yang et al. (2016)	aMCI *N* = 25	I : 67.8 ± 9.7 54.5% (F) C : 67.1 ± 9.5 42.9% (F)	I: memory training (verbal and visual association strategies and other strategies to improve memory) C: Yoga training	12 wk 60' session 1x/wk + 12' daily homework	12 wk GM sMRI: *N* = 25 ^1^H-MRS* N = 17	• I ↑ GMV of the dorsal ACC; not hipp. • Cho compounds ↓ after I in hipp. only. • No effect of I on GABA, Glx, NAA/NAAG met.	8.0 (50%)
Na et al. (2018)	HOA *N* = 10 SMI *N* = 6 MCI *N* = 10	HOA: 60.6 ± 5.3 90% (F) SMI: 62.2 ± 4.0 66.7% (F) MCI: 65.3 ± 4.0 80% (F)	I: CCT (attention, executive function, memory, calculation, visuospatial function, motor skills, problem-solving, and working memory domains)	12 wk 40' session 2x/wk	12 wk Glc met. im. & GM sMRI & DTI: *N* = 26	• I improved language and attention/psychomotor speed in HOA. • No effect of I on cognitive outcomes for SMI. • I improved TMT-B and W-B, memory, and executive-function in MCI. • For MCI participants, I ↑ activation for Glc imaging data in left ant. ins., left ACC and right lat. temp. cortex, ↑ cortical thickness in rostral ACC, and ↑ FA in many regions including the ACC. No effect of I on imaging outcomes for HOA and SMI.	8.0 (50%)
Vermeij et al. (2016)	aMCI *N* = 18 HOA *N* = 23	aMCI: 68.4 ± 6.3 22.2% (F) HOA: 70.1 ± 5.4 43.5% (F)	I: CCT (working memory domain)	5 wk 45' session 5x/wk	3 mo after the end of I GM sMRI and WMH im.: *N* = 31	• I improved digit span and spatial span for MCI and HOA (maintained at 3 mo followup) and figural fluency (RFFT). • Global brain and hipp. atrophy associated with participants' performance on neuropsychological outcomes. Higher WMH lesions associated with increased spatial span backward performance at the 3 mo followup.	8.0 (50%)
Feng et al. (2018)	aMCI *N* = 25	I1: 69.63 ± 3.54 50% (F) I2: 72.13 ± 3.56 50% (F) C: 68.13 ± 2.80 55.6% (F)	I1: CT on memory, reasoning,problem- solving ability, and visual-spatial reading skills (MDCT) I2 : reasoning (SDCT) C: passive control	12 wk 1 h session 2x/wk	12 wk GM sMRI: *N* = 23 rs-fMRI: *N* = 25	• ≠ between I1, I2, and C for GMV of mid. front., sup. par. lobule, inf. temp., fusiform gyrus, and ventral V3 areas. • Within I1 : Reho ↑ for front. and both ↑ and ↓ for temp. and occ. areas; I2 : Reho ↑ for temp. and occ., and both ↑ and ↓ in front. areas; C : Reho ↑ for temp. and occ. and ↓ for front. areas.	7.0 (44%)
Hohenfeld et al. (2017)	prAD *N* = 10 HOA *N* = 16 SH *N* = 4	HC: 63.5 ± 6.7 44% (F) prAD: 66.2 ± 8.9 20% (F) SH: 64.8 ± 9.5 25% (F)	I: neurofeedback training (real-world footpath encoding) C: sham feedback	Pre-session to encode the footpath followed by 3 days of training	Post training GM sMRI and rt-fMRI: *N* = 30	• Improvement after I within HOA and prAD for visuospatial memory performance and within HOA for the WMS backward digit-span task and in MoCa. No ≠ for SH. • Activation of the target region left PH gyrus for HOA and prAD during I, but no ≠ over the course of the I for PSC. No activation of left PH gyrus and no ≠ over the course of the I for PSC for SH. • FC modified during I for left PH gyrus and right PCu for HOA and for right PCu for prAD (GCA). • ↑ of GMV in right PCu and right sup. med. front. gyrus in prAD and HOA.	7.0 (44%)

↔, between; ↓, decrease; ≠, different; ↑, increase; ^1^H-MRS, proton magnetic resonance spectroscopy; ACC, anterior cingulate cortex; ACER, Addenbrooke's cognitive examination-revised; AD, Alzheimer's disease; aMCI, amnestic mild cognitive impairment; Ant, anterior; AxD, axial diffusivity; BC, betweenness centrality; Bilat, bilateral; BVMT-R, brief visuospatial memory test-revised; C, control condition; CCT, computerized cognitive training; Cer, cerebellum; Cing, cingulate; Cingu, cingulum; CFT, Rey-Osterrieth complex figure test; COWAT, controlled oral word association test; Cu, cuneus; CWST, color word stroop test; Cho, choline; DTI, diffusion tensor imaging; EEG, electroencephalography; F, female; FA, fractional anisotropy; FC, functional connectivity; Front, frontal; GABA, gamma-aminoButyric acid; Glc, glucose; Glx, glutamate-glutamine; NAA/NAAG, N-acetyl aspartate and N-acetylaspartyl-glutamate; GCA, granger causality analysis; GDS, geriatric depression scale; GM sMRI, gray matter structural magnetic resonance imaging; GMV, gray matter volume; Hipp, hippocampus; HOA, healthy older adults; HVLT-R, Hopkins verbal learning test-revised; I, intervention; Im, imaging; Inf, inferior; Ins, insula; Lat, lateral; M, male; MCI, mild cognitive impairment; MD, mean diffusivity; Med, medial; Met, metabolism; Mid, middle; MMSE, mini-mental state examination; Mo, months; MoCa, Montreal cognitive assessment; MRI, magnetic resonance imaging; Occ, occipital; Par, parietal; PCC, posterior cingulate cortex; PCu, precuneus; PH, parahippocampal; Post, posterior; prAD, prodromal AD; PSC, percent signal change; RBANS, repeatable battery for the assessment of neuropsychological status; Reho, regional homogeneity; RD, radial diffusivity; rs-fMRI, resting state functional magnetic resonance imaging; rt-fMRI, real-time functional magnetic resonance imaging; SMC/SMI, subjective memory complaints/impairment; SH, sham condition for healthy older adults; Sup, superior; SUT, strategy use task; Temp, temporal; t-fMRI, task functional magnetic resonance imaging; Th, thalamus; TMT-B and W-B, trail-making test—black and white; V3, visual cortex area V3; Wk, week; WMS, wechsler memory scale.

*We only reported the minimum number of participants for 1H-MRS GABA data. Number of participants for ^1^H-MRS Glx, Cho, and NAA/NAAG vary around 20 participants.

Various CT were used, computerized (*n* = 5) or non-computerized (*n* = 8), uni- or multi-domain and aimed at improving domains such as memory, attention or executive functions. Over the five computerized CT, four targeted multiple cognitive domains (Barban et al., 2017; Na et al., 2018; Li et al., 2019; Zhang et al., 2019) while only one focussed on solely improving working memory (Vermeij et al., 2016). Among the eight non-computerized CT, two used multi-domain CT (Ciarmiello et al., 2015; Park et al., 2019), one included both multi-domain and uni-domain (Feng et al., 2018), and five tested single domains (Belleville et al., 2011; Yang et al., 2016; Hohenfeld et al., 2017; Simon et al., 2018; Youn et al., 2019). The uni-domain CT targeted memory (Belleville et al., 2011; Yang et al., 2016; Simon et al., 2018), reasoning enhancement (Feng et al., 2018), metamemory (introspective knowledge of one's own memory capabilities) (Youn et al., 2019), and neurofeedback (real-time feedback from brain activity in order to reinforce healthy brain function through operant conditioning; Hohenfeld et al., 2017; Sitaram et al., 2017).

##### 3.2.3.1. Description of the effect of CT on brain structure

Two studies reported results about participants with nCI using multiple neuroimaging biomarkers: null results seemed to be prevalent after CT (4/5) with or without a comparison to a sham intervention (Figure 2).

Ten weeks of metamemory training (see Section 3.2.3) affected brain morphology in SCI participants, as compared to general education on memory (Youn et al., 2019). A decrease of WM mean diffusivity was observed, usually associated with increased myelination and axon density, while GMV was not impacted (Youn et al., 2019). Conversely, a 12 weeks multi-domain computerized CT did not affect WM nor integrity of GMV in SCI participants, however no sham condition was examined (Na et al., 2018). For patients with CI—no matter what the type of neuroimaging biomarker considered and for trials with sham interventions—positive results outweighed null results. This was further corroborated by publications not including a sham intervention (positive results: 17/21; Figure 2). All trials examining the effect of multiple CT on brain morphology presented positive results, especially on GMV (Table 5). For participants with MCI, GMV was increased and WM integrity preserved by 12 weeks of computerized CT targeting multiple domains (Na et al., 2018).

For aMCI, CT increased GMV, though brain regions affected differed between trials, most likely due to the differences in cognitive demand. Twelve weeks of multi-domain computerized CT increased GMV in the right angular gyrus (Zhang et al., 2019), a brain region associated with visuospatial attention that recently emerged as a cross-modal hub (Seghier, 2013). Differences of GMV in the middle frontal gyrus, superior parietal lobule, inferior temporal gyrus, fusiform gyrus, and visual cortex were observed in participants receiving 12 week of multi-domain CT (Feng et al., 2018). These structures have been shown to be involved in attention, visuospatial perception and recognition processes (Kanwisher et al., 1997; Yantis et al., 2002; Scheff et al., 2011; Japee et al., 2015). Eventually, 12 weeks of memory training increased GMV in the ACC, a region implicated in several complex cognitive functions such as decision-making; it did not however impact HV (Yang et al., 2016).

For prodromal AD participants, GMV was increased after a real-time neurofeedback training (3 days) in the right precuneus and superior medial frontal gyrus area (Hohenfeld et al., 2017), two regions involved in memory and decision-making (Rushworth et al., 2004; Cavanna and Trimble, 2006). Interestingly, change in GMV for aMCI could be associated with improvements in cognition. Global brain and hippocampus atrophy were related to cognitive performance after 5 weeks of computerized CT targeting working memory (Vermeij et al., 2016).

##### 3.2.3.2. Description of the effect of CT on brain function and metabolism

Only one study reported a lack of effect of CT on brain glucose metabolism in participants with nCI after 12 weeks of multi-domain computerized CT (Table 5). It should be mentioned that the sample size was reduced (*n* = 6), and that no sham intervention was included (Na et al., 2018).

For participants with CI, more data were available (k = 14). All studies without sham condition reported positive results on brain function and metabolism (k = 3) as did a majority of trials with sham procedure (7/11; Table 5). Null results were related to brain metabolism only. For participants with MCI, regional activity measured through rs-fMRI was increased bilaterally in temporal poles, insular lobes, and left parahippocampal gyrus after 6 months of multi-domain computerized CT (Li et al., 2019). These modifications were coherent with the changes observed in neuropsychological outcomes. A memory gain was associated with the modifications in temporal lobes and parahippocampal activities. Moreover, activation in the insular lobes could be linked to the gain of visual and semantic memory performances (Li et al., 2019).

Brain metabolism was affected for MCI participants and PET-FDG data showed focal activation in the insula, ACC and temporal cortex after multi-domain CT (Na et al., 2018).

For aMCI participants, 3 months of multi-domain computerized CT induced widespread FC changes (measured with rs-fMRI) over multiple brain regions, notably by increasing connectivity of the posterior area of the DMN (Barban et al., 2017). When focussing on local measures of FC it appeared that 12 weeks of multi or uni domain CT increased regional connectivity in the inferior frontal and precentral gyri (Feng et al., 2018).

Task-related brain activity was also modified by CT for aMCI participants. Two weeks of mnemonic CT increased the functional activation (fMRI) associated with memory encoding in the left anterior temporal lobe (Simon et al., 2018). This increase was consistent with improvement of the cognitive processes targeted by this training. Areas of the temporal cortex involved in social cognition and face processing showed increased activity while participants improved on face-naming tasks (Simon et al., 2018). Six weeks of CT of episodic memory modified brain functional activation (fMRI) as well (Belleville et al., 2011). This CT recruited distinct brain regions during encoding and retrieval tasks: increased activation was observed in parietal, temporal, and frontal areas as well as in insula, basal ganglia, and cerebellum during memory encoding while activation in parietal, frontal, and temporal cortices, as well as in the PCC and insula was observed during memory retrieval (Belleville et al., 2011). CT diminished the differences observed between HOA and aMCI participants prior to the intervention; it was interpreted as a rehabilitation of encoding activity in aMCI participants (Belleville et al., 2011). Eventually, a real-time neurofeedback CT modified the activation (fMRI) in the parahippocampal area as well as the FC of the precuneus for patients in the prodromal phase of AD (Hohenfeld et al., 2017).

Brain metabolism also seemed modified by CT for aMCI participants. Four months of multi-domain CT changed glucose metabolism in frontal, temporal, occipito/temporal, ACC and basal ganglia areas (Ciarmiello et al., 2015). Conversely, another trial administered during 12 weeks and using a CT mainly focussed on improving memory, frontal lobe function, and orientation had no effect on glucose metabolism (Park et al., 2019).

The effect of CT on other metabolites (choline compounds, GABA, Glx, and NAA-NAAG) was also assessed in a 12 weeks trial, showing that memory training decreased the choline-containing compounds in the hippocampus of aMCI patients without any effect on other metabolites (Yang et al., 2016).

#### 3.2.4. Multidomain intervention

Nine studies evaluated the impact of MD interventions on at-risk for AD subjects (Table 8). Global quality of MD interventions was good (Table 2). Participants were recruited from 70 to 78 years old on average with 31–73% of females. Intervention duration was between 12 weeks and 6 years. Frequency of MD interventions' domains, without considering domains focussed on nutrition, ranged from less than five sessions per year to three sessions per week, and sessions ranged from 20 to 120 min. MD interventions combined: PE, CT, and music therapy (*n* = 1; Train the Brain Consortium, 2017); PE with low or high cognitively demanding tasks (*n* = 1; Anderson-Hanley et al., 2018); resistance training with computerized CT (*n* = 1; Broadhouse et al., 2020); nutritional counseling, PE, CT and management of metabolic and vascular risk factors (*n* = 2; Stephen et al., 2019, 2020); CT, PE, and nutritional advice with or without ω-3 intake (*n* = 1; Delrieu et al., 2020); CT, counseling (mediation training, cognitive behavioral therapy, education regarding the Mediterranean diet, exercise, stress reduction strategies and sleep hygiene) and neurofeedback (*n* = 1; Fotuhi et al., 2016); ω-3 intake, aerobic exercise and cognitive stimulation (*n* = 1; Köbe et al., 2016); cardiovascular risk factors management using lifestyle advice and medical interventions (*n* = 1; van Dalen et al., 2017). The two publications from Stephen and al. were related to the Finnish Geriatric Intervention Study to Prevent Cognitive Impairment and Disability (FINGER) trial (Ngandu et al., 2015).

**Table 8:** Multidomain (MD) preventive interventions.

**References**	**Population(s)**	**Mean age (sd) Gender (F)**	**Intervention description**	**Intervention length/freq**.	**Imaging data at follow up**	**Main findings**	**Note**
Train the Brain Consortium (2017)	MCI *N* = 113	74.5 ± 4.6 48.7% (F)	I: physical, CT, and music therapy C: usual lifestyle	7 mo CT : 2 × 1 h session 3x/wk Musical activity: 1 h session 1x/wk PE: 1 h session 3x/wk	7 mo Perf MRI and GM sMRI: *N* = 70 t-fMRI (visuo- spatial attention task): *N* = 50	• I improved cognition (ADAS-cog) • No effect of I on hipp vol. nor on PH vol. • I ↑ CBF in PH area; not hipp. • BOLD signal ↑ within C.	14.0 (88%)
van Dalen et al. (2017)	High systolic blood pressure *N* = 126	I: 77.3 ± 2.6 53% (F) C: 77.1 ± 2.4 53% (F)	I: multidomain cardiovascular intervention, lifestyle advice, eventual drug treatment for hypertension, dyslipidemia, diabete C: usual care	6 years session 4x/mo	6 years WMH im.: *N* = 126	• No effect of I on WMH • No effect of I on lacunar infarcts development • I effect at baseline	12.0 (86%)
Stephen et al. (2019)	Increased risk of dementia (CAIDE) *N* = 132	I: 70.3 42.6% (F) C: 69.8 51.5% (F)	I: NA, PE, CT, and management of metabolic and vascular risk factors C: regular health advice	2 years NA: 9 to 1–3 session PE: 30–60' session 2-4x/wk to 2–3x/wk. CT: 10–15' individual CCT 3x/wk + 10x 60–90' group training session Management risk factors: 10 session	2 years GM sMRI: *N* = 112	• No effect of I on total and hipp. brain vol and AD signature areas cortical thickness, nor on WM lesions • Participants with higher cortical thickness on AD signatures areas benefit more from I on processing speed.	13.0 (81%)
Delrieu et al. (2020)	Older adults with SMC, limitation in one IADL, or slow gait speed *N* = 67	76.37 ± 4.23 73% (F)	I: MD intervention (CT, demonstrations about PE, NA) with or without ω-3 C: no MD	12 mo MD : 2 h session (1 h CT, 45' PE demos, 15' NA) h session/mo + 2 h session at 12 mo. ω-3: 400 mg DHA + max. 112.5 mg EPA daily	12 mo: Glc met. im.: *N* = 57	• No effect of I on meta-ROI (PCC, angular gyrus, temp. areas) global SUVR at 6 or 12 mo. • No effect of I on cognitive composite score at 6 or 12 mo. • For voxel-wise exploratory analysis, ↑ metabolism at 6 mo in right hipp, right post. cing, left post. PH areas, and right ins. cortex for I compared to C. • No effect of I at 12 mo.	13.0 (81%)
Broadhouse et al. (2020)	MCI *N* = 84	69.5 ± 6.6 69% (F)	I1 : PRT+CCT I2 : PRT+SHAM I3 : CCT+SHAM C : SHAM+SHAM	6 mo 90' session 2–3x/wk	6 mo GM sMRI: *N* = 79 rs-fMRI: *N* = 72	• I2 improved cognition (ADAS-cog) and executive functions, compared to C 12 mo after intervention has ended (18 mo). • Long-term effect, at 18 mo, of I1 and I2 on atrophy rate of left hipp compared to C. Short-term effect of I1 and I2 at 6, but not at 18 mo on left PCC cortical thickness. • Combined I1+I2 ↑ PCC and hipp FC at 18 mo compared to combined I3+C, but no effect of I1, I2 or I3 alone vs. C.	11.0 (69%)
Stephen et al. (2020)	Increased risk of dementia (CAIDE) *N* = 60	I: 70.02 ± 4.2 41% (F) C: 69.55 ± 3.9 50% (F)	See Stephen et al. (2019)	See Stephen et al. (2019)	2 years DTI: *N* = 60	• I improved NTB score. • FA ↓ after I. • In C, FA change correlated to NTB total score change; RD change negatively correlated to changes in NTB total score, NTB executive function and memory scores.	11.0 (69%)
Köbe et al. (2016)	aMCI *N* = 22	I: 70 ± 7.2 31% (F) C : 70 ± 5.2 44% (F)	I: ω-3 intake, AE , cognitive stimulation C: ω-3 intake, non AE training	6 mo ω-3: 2,200 mg/day AE : 45' session 2x/wk CT: 13x 90' session	6 mo GM sMRI: *N* = 20	• No effect of I on executive function, memory, sensorimotor speed, and attention. • GMV ↑ after I in front., par. and cing. cortex areas. • Decrease in homocysteine concentration associated with higher mean GMV of in the mid. front. cortex.	10.0 (62%)
Anderson-Hanley et al. (2018)	MCI/screened for MCI *N* = 111	78.1 ± 9.9 66% (F)	I1: PE (pedaling) + low cognitive demand task I2: PE + high cognitive demand task I3: videogame C: pedal-only*	6 mo 20' session 2x/wk to 45' session 3x/wk	6 mo GM sMRI: *N* = 8	• Within I1 and I2 groups, improvement on executive function at 6 mo; I1 improved earlier at 3 mo. • Greater exercise dose associated with ↑ of PFC and ACC volumes, and greater memory performance associated with ↑ DLPFC volume.	9.0 (56%)
Fotuhi et al. (2016)	MCI *N* = 127	70.69 ± 10.53 63% (F)	Personalized CCT, counseling/brain coaching, neurofeedback training.	12 wk CT+Neurofeedback: 2 h session 1x/wk Counseling: 1 h session 1x/wk	12 wk GM sMRI: *N* = 17	• Improvement on cognition (≥ 3/10 areas of cognition). • Preservation or growth of hipp. vol. for 12/17 participants.	3.0 (23%)

↓, decrease; ↑, increase; ACC, anterior cingulate cortex; AD, Alzheimer's disease; ADAS-cog, Alzheimer's disease assessment scale-cognitive subscale; AE, aerobic exercise; aMCI, amnestic mild cognitive impairment; BOLD, blood oxygen level dependant; C, control condition; CAIDE, cardiovascular risk factors aging and incidence of dementia; CBF, cerebral blood flow; CCT, computerized cognitive training; CCT+SHAM, computerized cognitive training and sham exercise; Cing, cingulate; CT, cognitive training; DLPFC, dorsolateral prefrontal cortex; DTI, diffusion tensor imaging; F, female; FA, fractional anisotropy; Front, frontal; Glc, glucose; GM sMRI, gray matter structural magnetic resonance imaging; GMV, gray matter volume; Hipp, hippocampus; I, intervention; IADL, instrumental activity of daily living; Im, imaging; Ins, insula; M, male; MD, multidomain; MCI, mild cognitive impairment; Met, metabolism; Mo, months; NA, nutritional advice; Mid, middle; MRI, magnetic resonance imaging; NTB, neuropsychological test battery; Par, parietal; PCC, posterior cingulate cortex; PE, physical exercise; Perf, perfusion imaging; PFC, prefrontal cortex; PH, parahippocampal; Post, posterior; PRT+CCT, progressive resistance and computerized cognitive training; PRT+SHAM, progressive resistance and cognitive sham training; RD, radial diffusivity; ROI, region Of interest; rs-fMRI, resting state functional magnetic resonance imaging; RT, resistance training; SHAM+SHAM, sham physical and sham cognitive training; SMC, spontaneous memory complaint; SUVR, standard uptake value ratio; Temp, temporal; t-fMRI, task functional magnetic resonance imaging; Wk, week; WM, white matter; WMH, white matter hyperintensities.

*The pedal-only group was recruited through the Cybercycle Study (Anderson-Hanley et al., 2012).

##### 3.2.4.1. Description of the effect of MD intervention on brain structure

The same number of studies (*n* = 4) examined the effect of MD interventions on populations with or without CI (Figure 2). For participants with nCI, results were mostly null (Figure 2) whatever the imaging biomarker considered. All studies included a sham intervention group (Figure 2). The impact of MD interventions on brain morphology was limited: only one study reported an effect on WM structure, while three null results were observed on GM and WM structures (Table 5).

For subjects with an increased CAIDE score, MD intervention administered during 2 years (FINGER trial) led to a decrease of FA (Stephen et al., 2020). FA, which measures the degree of anisotropy of water molecules' diffusion, is often decreased in AD patients in multiple WM areas (Sexton et al., 2011; Teipel et al., 2014). This decrease has been linked to a breakdown in structured myelin (Stricker et al., 2009). It was thus surprising to observe such a decrease of FA after intervention. The authors argued that this decrease was not associated with cognitive decline, and that FA increase, probably due to axonal swelling and astrocytic hypertrophy, had previously been observed in the early stage of AD. Thus, the decrease in FA seems an indication of WM integrity (Stephen et al., 2020). In another publication related to the FINGER cohort, no effect on total GMV, HV, cortical thickness in AD signature areas nor on WM lesions were observed (Stephen et al., 2019). A 6 years MD intervention in elderly with hypertension (PreDIVA trial) did not significantly impact the progression of WMH (van Dalen et al., 2017).

It should be mentioned that subjects with higher baseline cortical thickness in the FINGER trial benefited more from intervention (Stephen et al., 2019) and that the effect of intervention was also more important in participants with more WMH lesions at baseline in the PreDIVA trial (van Dalen et al., 2017). Both studies thus suggest that particular care should be taken when considering the population selected for MD intervention either with fewer structural brain changes or with high WMH volumes. In contrast to nCI, results for participants with CI were mostly all positive no matter what the type of neuroimaging biomarker considered, and all studies except for one included sham interventions (Table 5). Only one null result was reported concerning GM structure (Table 5; Train the Brain Consortium, 2017).

After 6 months of MD intervention, exercise effort was associated with increased prefrontal cortex (PFC) and ACC volume in MCI participants (Anderson-Hanley et al., 2018). HV was preserved by 12 weeks of MD intervention (Fotuhi et al., 2016). Moreover, hippocampal atrophy rate was reduced 12 months after a 6 months MD intervention (Broadhouse et al., 2020). Cortical thickness in the PCC was significantly increased by this intervention and long-term benefits on cognition were obtained (Broadhouse et al., 2020). Conversely, it should be mentioned that a 7 months MD intervention had no effect on hippocampal and parahippocampal volumes while improving cognition (Train the Brain Consortium, 2017).

For participants with aMCI, 26 weeks of MD intervention increased GMV in frontal, parietal, and cingulate cortex areas (Köbe et al., 2016).

##### 3.2.4.2. Description of the effect of multidomain intervention on brain function, perfusion, and metabolism

Few studies reported the effect of MD on brain function, perfusion or metabolism (Table 5). All studies included a sham intervention.

For participants with nCI, a 6 months MD intervention (MAPT trial, see Section 3.2.4) increased glucose metabolism in limbic areas (right hippocampus, right posterior cingulate, left posterior parahippocampal gyrus; Delrieu et al., 2020). This effect vanished after 12 months, potentially because the frequency of interventions decreased after the first 2 months (Delrieu et al., 2020).

For participants with MCI, 7 months of MD intervention increased CBF in the parahippocampal area, possibly indicating a better perfusion state (Train the Brain Consortium, 2017). Moreover, brain regions involved in a visuo-spatial attention task showed preserved blood oxygen level-dependent (BOLD) signal (fMRI) after intervention, suggesting that the neural efficiency was preserved (Train the Brain Consortium, 2017).

#### 3.2.5. Impact on cognition

Some of the studies that report an effect of intervention on neuroimaging biomarkers also assess the effect on cognition. The number of results reported for studies evaluating cognition on nCI participants was limited and we could not determine whether these results reinforced the observations made on neuroimaging biomarkers (Supplementary Table 3). For studies that reported an effect of PE, nutrition or CT on neuroimaging biomarkers for CI participants, an effect of the interventions on cognition was also detected in most cases (Supplementary Table 3). Studies providing positive neuroimaging results after MD intervention provided mixed results on cognition (Supplementary Table 3).

### 3.3. Target brain regions for interventions

#### 3.3.1. Preferential target regions

Of the 43 publications assessing the impact of the various interventions, 10 focused on specific brain regions preferentially targeted by AD physiopathology in addition to whole brain analysis and 10 focused on specific regions only. The hippocampus was preferentially studied (*n* = 17), followed by the ACC (*n* = 3), the PCC (*n* = 2), the parahippocampal area (*n* = 2), the PCC/Precuneus area (*n* = 1), the whole DMN (*n* = 1), and the prefrontal cortex (*n* = 1).

#### 3.3.2. Effect of all type of interventions on brain regions

**Figure 3:**
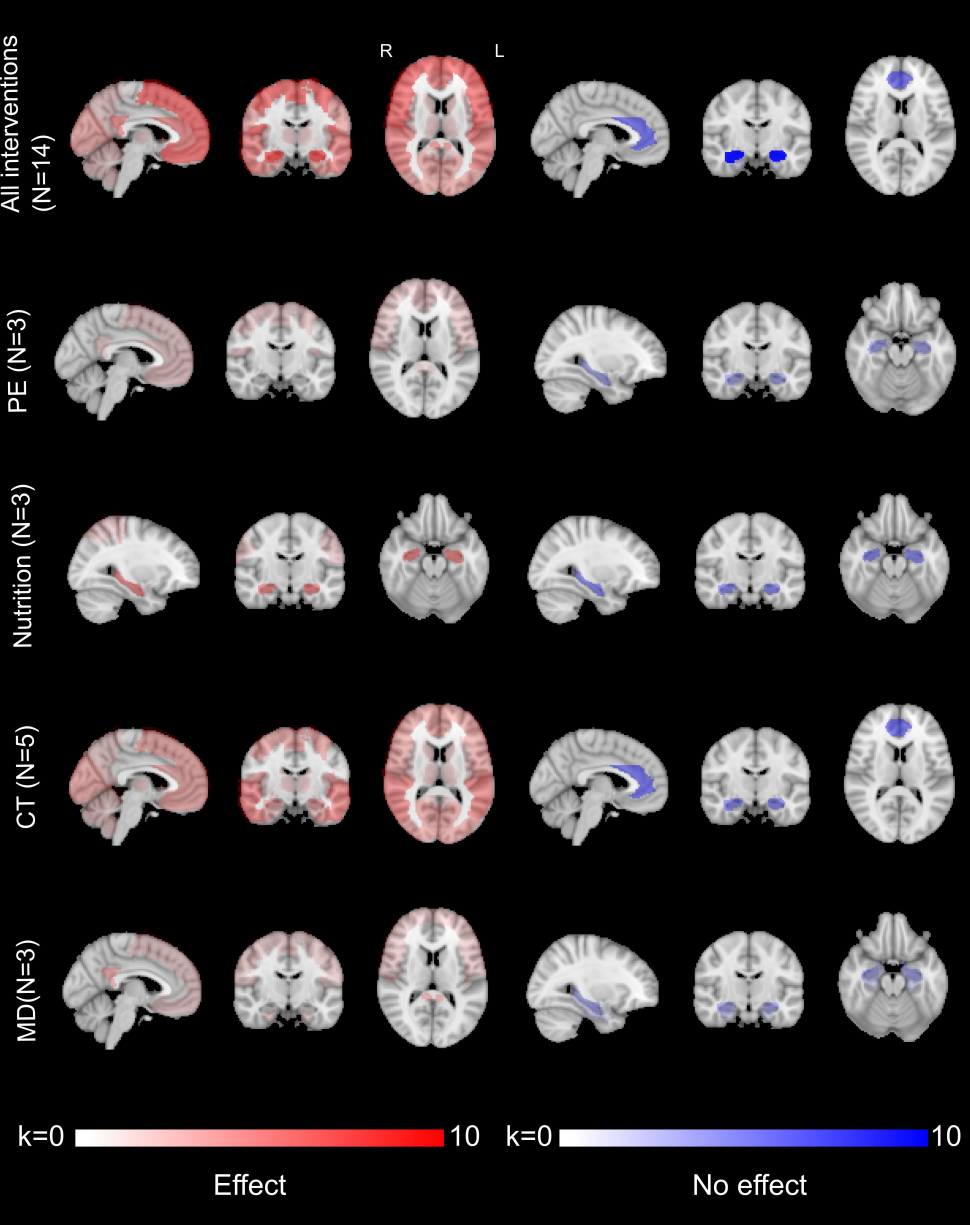
Brain regions affected by the interventions for participants with CI, for all imaging modalities and after direct comparison to a sham intervention. Color grading is used to display the number of results (k), positive (effects of interventions on brain regions through whole-brain or region-targeted analyses; on the left in red) or null (lack of effect of interventions after brain regions have been specifically targeted; on the right in blue), over brain regions. Please refer to the Supplementary Figure 2 for the count of results (k) for each named regions. CT, cognitive training; k, number of results; MD, multidomain intervention.

All studies we selected to evaluate the impact of interventions on specific brain regions included participants with CI and used a sham intervention group as a control. All obtained good to high quality ratings (Table 9). When considering all types of interventions together, the hippocampus and frontal areas were the brain regions that appeared the most impacted by the interventions (k = 5 and 4, respectively; Figure 3, Table 9). While a larger amount of null results was observed for the hippocampus, it should be mentioned that more than half of them concerned analyses relative to structural biomarkers. Interestingly, positive results were distributed between neuroimaging modalities including metabolism, function, and structure (Table 9). For frontal regions, positive results were spread between brain metabolism, structure, and function.

**Table 9:** Effects of interventions on brain regions (see Figure 3) for each modality.

**Region**	**All (*****N*** **= 14)**		**PE (*****N*** **= 3)**		**Nutrition (*****N*** **= 3)**		**CT (*****N*** **= 5)**		**MD (*****N*** **= 3)**	
	**Effect**	**No effect**	**Effect**	**No effect**	**Effect**	**No effect**	**Effect**	**No effect**	**Effect**	**No effect**
ACC (*N* = 3)	Perf. MRI (k = 1, q = 69%) Glc met. im. (k = 1, q = 62%) GM sMRI (k = 1, q = 50%)	Ot. met. im. (k = 4, q = 50%)	Perf. MRI (k = 1, q = 69%)	k = 0	k = 0	k = 0	Glc met. im. (k = 1, q = 62%) Perf. MRI(k = 1, q = 50%)	Ot. met. im. (k = 4, q = 50%)	k = 0	k = 0
CN (*N* = 1)	Glc met. im. (k = 1, q = 62%)	k = 0	k = 0	k = 0	k = 0	k = 0	Glc met. im. (k = 1, q = 62%)	k = 0	k = 0	k = 0
Cer (*N* = 1)	fMRI (k = 1, q = 69%)	k = 0	k = 0	k = 0	k = 0	k = 0	fMRI (k = 1, q = 69%)	k = 0	k = 0	k = 0
Front (*N* = 4)	fMRI (k = 2, q = 72%) GM sMRI (k = 1, q = 62%) Glc met. im. (k = 1, q = 62%)	k = 0	fMRI (k = 1, q = 75%)	k = 0	k = 0	k = 0	fMRI (k = 1, q = 69%) Glc met. im. (k = 1, q = 62%)	k = 0	GM sMRI (k = 1, q = 62%)	k = 0
GP (*N* = 1)	fMRI (k = 1, q = 69%)	k = 0	k = 0	k = 0	k = 0	k = 0	fMRI (k = 1, q = 69%)	k = 0	k = 0	k = 0
Hipp (*N* = 8)	GM sMRI (k = 2, q = 81%) fMRI (k = 2, q = 69%) Ot. met. im. (k = 1, q = 50%)	GM sMRI (k = 4, q = 72%) Perf. MRI (k = 2, q = 78%) Ot. met. im. (k = 2, q = 50%) WM sMRI (k = 1, q = 69%)*	k = 0	GM sMRI (k = 1, q = 80%) Perf. MRI (k = 1, q = 69%)	GM sMRI (k = 2, q = 81%) fMRI (k = 1, q = 69%)	GM sMRI (k = 1, q = 69%) WM sMRI (k = 1, q = 69%)*	fMRI (k = 1, q = 69%) Ot. met. im. (k = 1, q = 50%)	Ot. met. im. (k = 2, q = 50%) GM sMRI (k = 1, q = 50%)	k = 0	Perf. MRI (k = 1, q = 88%) GM sMRI (k = 1, q = 88%)
Ins (*N* = 1)	fMRI (k = 1, q = 69%)	k = 0	k = 0	k = 0	k = 0	k = 0	fMRI (k = 1, q = 69%)	k = 0	k = 0	k = 0
Occ (*N* = 2)	fMRI (k = 1, q = 69%) Glc met. im. (k = 1, q = 62%)	k = 0	k = 0	k = 0	k = 0	k = 0	fMRI (k = 1, q = 69%) Glc met. im. (k = 1, q = 62%)	k = 0	k = 0	k = 0
PCC (*N* = 3)	GM sMRI (k = 2, q = 66%) Perf. MRI (k = 1 ,q = 69%)	k = 0	Perf. MRI (k = 1, q = 69%)	k = 0	k = 0	k = 0	k = 0	k = 0	GM sMRI (k = 2, q = 66%)	k = 0
PCu (*N* = 1)	fMRI (k = 1, q = 69%)	k = 0	k = 0	k = 0	k = 0	k = 0	fMRI (k = 1, q = 69%)	k = 0	k = 0	k = 0
PH (*N* = 2)	Perf. MRI (k = 1, q = 88%) fMRI (k = 1, q = 69%)	GM sMRI (k = 1, q = 88%)	k = 0	k = 0	k = 0	k = 0	fMRI (k = 1, q = 69%)	k = 0	Perf. MRI (k = 1, q = 88%)	GM sMRI (k = 1, q = 88%)
Par (*N* = 2)	fMRI (k = 1, q = 69%) GM sMRI (k = 1, q = 62%)	k = 0	k = 0	k = 0	fMRI (k = 1, q = 69%)	k = 0	k = 0	k = 0	GM sMRI (k = 1, q = 62%)	k = 0
Temp (*N* = 3)	fMRI (k = 2, q = 69%) Glc met. im. (k = 1, q = 62%)	k = 0	k = 0	k = 0	k = 0	k = 0	fMRI (k = 2, q = 69%) Glc met. im. (k = 1, q = 62%)	k = 0	k = 0	k = 0
Th (*N* = 1)	fMRI (k = 1, q = 69%)	k = 0	k = 0	k = 0	k = 0	k = 0	fMRI (k = 1, q = 69%)	k = 0	k = 0	k = 0
Vent (*N* = 2)	GM sMRI (k = 1, q = 93%)	GM sMRI (k = 1, q = 69%)	k = 0	k = 0	GM sMRI (k = 1, q = 93%)	GM sMRI (k = 1, q = 69%)	k = 0	k = 0	k = 0	k = 0

For a set of k results, the mean quality q of the studies from which the results were reported was specified. Only results obtained after a direct comparison of intervention to a sham intervention for CI participants were examined. *Here DTI is used to assess hippocampus' mean diffusivity; in order to homogenize our terminology it is classified in the WM sMRI category although it is not WM that is evaluated. *N*, number of studies; k, number of results from all available studies; ACC, anterior cingulate cortex; CN, caudate nuclei; Cer, cerebellum; Front, frontal areas; CT, cognitive training; Glc, glucose; GM, gray matter; GP, globus pallidus; Hipp, hippocampus; Im, imaging; Ins, insula; Met, metabolism; MD, multidomain; MRI, magnetic resonance imaging; Occ, occipital areas; Ot, other; Par, parietal areas; PCC, posterior cingulate cortex; PCu, precuneus; PE, physical exercise; Perf, perfusion; PH, parahippocampal area; q, mean quality score; sMRI, structural MRI; Temp, temporal areas; Th, thalamus; Vent, ventricles; WM, white matter.

When considering each intervention individually, it appeared that some regions were particularly impacted by CT. Only CT had an effect on temporal (k = 3) and occipital (k = 2) areas. This was also the case for caudate nuclei, cerebellum, globus pallidus, insula, precuneus, and thalamus. Evidence for an effect of CT is however supported by only one study for these regions (Table 9).

Some brain regions were impacted by multiple interventions. Aside from nutritional interventions, multiple types of interventions affected frontal areas, even if only a limited number of studies described it (*n* = 4). Conversely, several types of interventions reported null results concerning the hippocampus, though few studies using nutritional or CT interventions reported positive impact.

## 4. Discussion

The aim of our review was to characterize the effects of preventive interventions on different modalities of neuroimaging biomarkers, for populations at-risk to develop AD and with or without CI.

**Figure 4:**
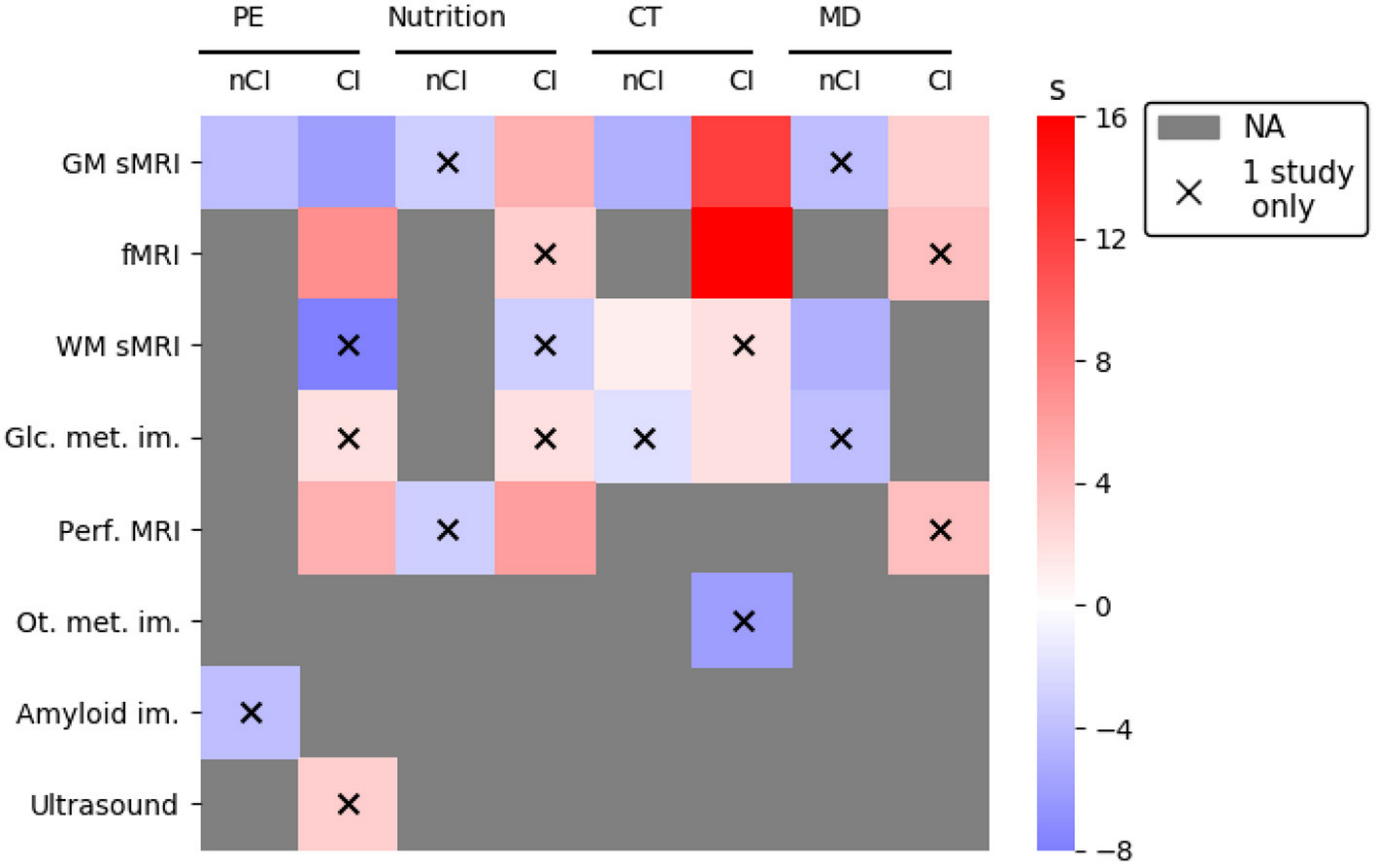
Synthetic representation of the effects of interventions on neuroimaging biomarkers. The effect, or lack of effect, of an intervention was evaluated through a composite “s” score integrating the number of results/studies, the quality of the studies, and the presence of a sham intervention to compare to (see Section 2). Scores with a red tint indicate an effect of the intervention, while scores with a blue tint indicate a lack of effect of intervention. Were not taken into account to generate this figure results from intervention intermediate timepoints (Delrieu et al., 2020: results at 6 months, Soininen et al., 2017) or followup measures (Broadhouse et al., 2020: results at 18 months), from a mixed sample of participants (Neth et al., 2020: ketone uptake and glucose metabolism), and studies reporting only results from correlation analyses (Vermeij et al., 2016; Anderson-Hanley et al., 2018; Hama et al., 2020), or analyzing specific subgroups within a clinical trial (Jernerén et al., 2015; Kaufman et al., 2021). CI, cognitive impairment; CT, cognitive training; fMRI, functional MRI; Glc, glucose; GM, gray matter; Im, imaging; MD, multidomain; Met, metabolism; MRI, magnetic resonance imaging; NA, no study available for the modality; nCI, no cognitive impairment; PE, physical exercise; Perf, perfusion; Ot, Other; sMRI, structural MRI; WM, white matter.

Globally, a positive impact was disclosed, whatever the intervention considered, on participants with CI. In most of the cases, interventions involving nCI participants reported no effect. An effect was detected for most neuroimaging modalities and interventions on participants with CI (Figure 4). Of all the modalities examined, functional imaging provides the most evidence of an effect of preventive interventions. This may indicate that this modality is particularly keen to detect brain alterations following preventive interventions. Only interventions using CT reported an effect on the temporal and occipital areas, while frontal areas were affected by all types of interventions except nutrition. Surprisingly, all types of interventions reported null results on the hippocampus region, though few nutritional or CT interventions reported a positive impact.

Multiple reviews are consistently reporting an effect of interventions on neuroimaging biomarkers for a large spectrum of cognitive profiles ranging from HOA to demented patients (ten Brinke et al., 2017; Haeger et al., 2019; van Balkom et al., 2020; Jensen et al., 2021). In this review, we observed that the effect of interventions differed according to cognitive profiles. Haeger et al. (2019) previously reported that the effect of PE varied on participants with different cognitive profiles (HOA vs. MCI or AD). Hippocampus cortical thickness was affected by PE for both groups, but interestingly PE affected the whole temporal lobe for HOA, whereas for AD/MCI subjects, the impact seemed concentrated on specific ROIs such as the hippocampus, the frontal lobe and the precuneus (Haeger et al., 2019).

Most reviews report that PE, CT or nutritional interventions affected the DMN, frontal or temporal areas. Our review concurred to the fact that most interventions affected the frontal cortex, a region involved in the cognitive decline associated with AD (Bayram et al., 2018; DeTure and Dickson, 2019). Furthermore, we noticed that many studies did not detect an effect of intervention on the hippocampus. A review examining the effect of PE on HOA or patients with mild AD, reported that only two out of seven studies detected an effect of PE on hippocampal volume (Frederiksen et al., 2018). We discuss this point further in the discussion.

The inconsistencies observed between studies could be explained by the heterogeneity of the populations and intervention protocols.

Age ranged between 61 and 81 years old for the included studies and the largest age difference between trials was observed for diet with a 16 years age difference. Age is the main risk factor for AD (Guerreiro and Bras, 2015). Older patients accumulate aged-associated alterations that may influence the impact of interventions and eventually explain discrepancies within a specific domain. Moreover, some risk factors between 20 and 60 become protective after 75 in a process called “reverse causality” indicating that age window for intervention is crucial (Kivipelto et al., 2018).

The proportion of females ranged between 20 and 100% (ten Brinke et al., 2015). CT had the most variable sample with a 70% difference in female proportion. Sex is an important factor that modulates the prevalence of AD risk factors but also the susceptibility to AD conferred by a given risk factor (Ferretti et al., 2018). For instance, women with APOE ϵ4 genotype are more susceptible to convert to AD than men with the same genotype, and socio-economic risk factors such as low level of education differ among men and women (Ferretti et al., 2018). Differences in sex sensibility to interventions could explain, at least partly, the discrepancies between trials (Podcasy and Epperson, 2016).

At-risk for AD is an extremely heterogeneous nosographic entity encompassing factors as diverse as education, blood pressure, BMI, total cholesterol, or physical activity. If each factor taken individually is clearly associated with an increased risk of developing AD it is not sure whether they are all impacted in the same way by interventions and might explain why trials on at-risk participants obtained, in the best cases, modest results. Risk factors, without additional markers of AD, might not be sufficient to select patients for interventions. In this review, we observed that interventions appear to be most effective in participants with CI. Impacts were limited in participants with risk factors for AD without objective cognitive impairment. It has been suggested for multidomain interventions that their effects might be the highest in healthy older adults (Rosenberg et al., 2020) and that interventions should be applied early, before the onset of substantial brain pathology and cognitive impairment (Solomon et al., 2021). However, it can prove difficult to identify individuals at risk for AD in the early stages of the disease when patients are still cognitively unimpaired and without accurate biomarkers for the disease. Participants at these stages may constitute a heterogeneous group and exhibit different progression profiles. Some will remain stable for a long time and/or never progress to AD. Not all participants may thus react in the same way to preventive interventions and not all may be optimal targets for preventive strategies. This may explain why the effect of interventions remains limited for the category of participants with risk factors for AD but without objective cognitive impairment. Selection might require additional markers of disease state, such as plasma biomarkers of amyloidosis (Lista et al., 2015), to further select participants already engaged in AD physiopathology or exhibiting stronger indications of the disease. It seems that there is a crucial balance between early at-risk participants, for which interventions would have no or limited impacts, and patients already engaged in the disease process for which appropriate interventions would have a clear benefit on cognition and neuroimaging biomarkers. Our interpretation is confirmed by results indicating that even if interventions seem to have no effect on global populations, they are efficient when specific subpopulations are considered. This is the case for the MAPT study in which participants with a CAIDE ≥ 6 or amyloid positive, thus indicating more at-risk or more engaged in AD physiopathology, could benefit more from the MD intervention (Andrieu et al., 2017).

Eventually, differences in interventions' design might be a source of heterogeneity. The duration of the intervention might be insufficient to bring impactful modifications to the brain. Similarly, intensity or frequency of the intervention might be too weak to bring significant changes. Conversely, very intense or frequent sessions might be counterproductive by decreasing protocol adherence. Eventually, one of the main hypotheses concerning MD interventions is that they were more efficient than unidomain ones (Kivipelto et al., 2018); this is not observed in our review. This could be explained if the optimal settings for each component were not met, or if the combination does not simply add up the individual effects. Alternative causes could be that complex multidomain interventions are difficult to adhere to, or that the positive effect of any component is somehow parasitized by any of the others.

Our review has several strengths. First, most of the studies included in the review are of good quality according to objective notation criteria already used by others. We reported studies' known biases when analyzing results, and proceeded to report results quantitatively. Second, a broad spectrum of imaging modalities was covered. Third, this review gave a large overview of the concurrent and separate effects of multiple interventions on the brain of older adults susceptible to converting to AD. Some limitations should however be considered. Populations were split into two categories, nCI or CI, without more subtle classifications. nCI participants gathered SCI together with cardiovascular risk factors. Similarly, CI regrouped MCI with more specific aMCI. It might have been inaccurate to pool together participants that react differently to interventions. We did not clarify whether the positive results reported for an intervention and a modality described concurrent or opposite effects (e.g., if for instance brain FC was decreased or increased). It is important to point out that we focused for this review on the PUBMED database which is public and easily accessible. However, we may have missed studies in other databases that could have been relevant to this review. Finally, the nosographic entity of AD has varied over time, and we may have missed studies relevant to our review when screening abstracts.

Several points should be emphasized in this review. Structural imaging of gray matter was the most studied modality, probably because it is very commonly acquired and presents homogenous acquisition and analysis methods. It seemed however less responsive to interventions. There is less consensus for other types of brain imaging biomarkers, which could partly explain the differences observed on these markers between studies. Early phases of AD might be associated with subtle brain alterations concerning metabolism or functionality, and could thus benefit from a development of the studies in these fields and an homogenization of the processes.

Furthermore, around 20% of the included studies assessed the effect of intervention by focussing on specific ROI known for their implication in AD such as the hippocampus. While this approach is founded on a strong biological assumption, it reduces the scope of analysis and is very dependent on the definition of the region (van Balkom et al., 2020). We noticed that the hippocampus was the region the most studied in this review. Interestingly, part of the studies failed to detect an effect of intervention on this structure, which is surprising as hippocampal atrophy can be used as an early factor of diagnosis in AD (Albert et al., 2011). Notwithstanding any methodological issue, different factors can explain this lack of effect. First, many null results relate to structural biomarkers. We hypothesize that interventions need to have a strong effect and to be implemented long enough in order to modify the hippocampus structure, which may not be the case for the interventions examined here. Furthermore, the changes brought by the intervention may not be visible at the standard 1 mm scale. We may also assume that interventions may modify specific hippocampal subfields that are affected differently in AD (La Joie et al., 2013; Baek et al., 2022), and that the measurement of the hippocampus as a whole leads to a noisy assessment of the intervention's effect.

At last, few studies met the criterion for statistical power. Scanning large samples of participants for brain images is often challenging, limited by logistic and financial issues. Future studies with increased sample size, power calculation, quality control procedures and control for multiple testing over brain image should be encouraged.

## 5. Conclusion

Our review indicates that preventive strategies involving PE, nutrition, CT, or MD interventions can change the brain of older adults with cognitive impairment. Frontal areas, which are known regions affected by neurodegenerative processes, were preferentially affected by a majority of interventions. Surprisingly, a large number of null results concerning the effect of interventions on the hippocampus were reported.

Taken together, results from multiple intervention trials all seem to indicate the importance of patient selection not only in terms of general characteristics as sex or age but also in terms of disease stage or risk factors. Deciphering the global and regional brain effect of each and combined interventions will help to better understand the interplay relationship between interventions, cognition, surrogate brain markers, and to better design primary and secondary outcomes for future preventive clinical trials. To conclude, this review seems to indicate that intervention may mitigate brain decay for participants at risk for AD and that have yet to develop dementia.

## Data availability statement

The original contributions presented in the study are included in the article/Supplementary material, further inquiries can be directed to the corresponding authors.

## Author contributions

LP and GB examined the contents of the studies included in this review. All authors contributed to manuscript writing, revision, and approved the submitted version.

## Funding

This work was publicly funded through ANR (the French National Research Agency) under the Investissements d'avenir programme with the reference ANR-16-IDEX-0006.

## Conflict of interest

The authors declare that the research was conducted in the absence of any commercial or financial relationships that could be construed as a potential conflict of interest.

## Publisher's note

All claims expressed in this article are solely those of the authors and do not necessarily represent those of their affiliated organizations, or those of the publisher, the editors and the reviewers. Any product that may be evaluated in this article, or claim that may be made by its manufacturer, is not guaranteed or endorsed by the publisher.

## Abbreviations

18F-AV-45: florbetapir
18F-FDG: 18F-fluorodeoxyglucose
Aβ: amyloid-beta
ACC: anterior cingulate cortex
AD: Alzheimer's disease
AHAD: American Heart Association diet
aMCI: amnestic MCI
APOE: apolipoprotein E
ASL: arterial spin labeling
BOLD: blood oxygen level-dependent
CAIDE: Cardiovascular Risk Factors Aging and Incidence of Dementia
CI: cognitive impairment
CBF: cerebral blood flow
CERAD: Consortium to Establish a Registry for Alzheimer's disease
CSF: cerebrospinal fluid
CT: cognitive training
DHA: docosahexaenoic acid
DMN: default mode network
DSC: dynamic susceptibility contrast
DTI: diffusion tensor imaging
EPA: eicosapentaenoic acid
FA: fractional anisotropy
FC: Functional connectivity
FLAIR: fluid attenuated inversion recovery
fMRI: functional magnetic resonance imaging
GABA: gamma-aminobutyric acid
Glx: glutamate-glutamine
GM: gray matter
GMV: gray matter
volume
HOA: healthy older adult
HV: hippocampal volume
MCI: mild cognitive impairment
MD: multi-domain
MMKD: modified Mediterranean-ketogenic diet
MMSE: mini mental state examination
MoCA: Montreal cognitive assessment
NAA-NAAG: N-acetyl aspartate and N-acetylaspartyl-glutamate
nCI: no cognitive impairment
NMR: nuclear magnetic resonance
PA: Physical activity
PCC: posterior cingulate cortex
PE: physical exercise
PET: positron emission tomography
PFC: prefrontal cortex
rs-fMRI: resting-state functional magnetic resonance imaging
SMC: subjective memory complaints
SMI: subjective memory impairment
SUVR: standard uptake value ratio
t-fMRI: task-based functional magnetic resonance imaging
WHO: World Health Organization
WM: white matter
WMH: white matter hyperintensity
ω3: omega-3.
