# Supplementary material for: Effects of preventive interventions on neuroimaging biomarkers in subjects at-risk to develop Alzheimer's disease: A systematic review
Source: Front Aging Neurosci. 2022 Nov 24;14:1014559. doi: 10.3389/fnagi.2022.1014559 (PMC9730537; doi:10.3389/fnagi.2022.1014559)
Supplement: Supplementary file 1 [file Data_Sheet_1.PDF]

SUPPLEMENTARY MATERIAL

PUBMED query: ((”elderly”[All Fields]) OR (”frail elderly”[All Fields]) OR (”risk factors”[All Fields]) OR (”mci”[All Fields]) OR (”alzheimer”[All Fields])) AND ((”pet”[All Fields]) OR (”brain imaging”[All Fields]) OR (”mri”[All Fields]) OR (”structural mri”[All Fields]) OR (”functional mri”[All Fields])) AND ((”training”[All Fields]) OR (”nutrition”[All Fields]) OR (”diet”[All Fields]) OR (”physical activity”[All Fields]) OR (”cognitive training”[All Fields]) OR (”cognitive stimulation”[All Fields]) OR (”exercise”[All Fields]))

1 TABLES

Table S1. Description of the included studies according to the PICO (Population, Intervention, Comparison, Outcomes) framework.

P	Older adults at risk for AD, that can 1) present risk factor for AD (e.g: APOE ϵ4), or/and 2) biomarkers for AD, and/or 3) subjective or cognitive impairment.
I	Physical exercise, nutritional intervention, cognitive training, multidomain interventions.
C	Comparisons were made between interventions and “sham interventions” groups. All types of sham interventions were considered for this review. When no sham intervention was provided in a study, the difference between pre and post intervention states was examined.
O	All outcomes derived from magnetic resonance neuroimaging techniques were examined.

Table S2. Studies excluded from the review after full-text review.

Study	Justification for not including the study in the review	Identified from
(Pantoni et al., 2017)	Vascular cognitive impairment	PUBMED query
(Hsu et al., 2018)	Vascular cognitive impairment	PUBMED query
(Tang et al., 2019)	Vascular cognitive impairment	PUBMED query
(Qi et al., 2018)	Specific type of intervention	PUBMED query
(Smart et al., 2016)	Specific type of intervention	PUBMED query
(Xia et al., 2019)	Specific type of intervention	PUBMED query
(Doniger et al., 2018)	Trial not completed	PUBMED query
(Sala-Vila et al., 2021)	Observational study	PUBMED query
(Um et al., 2020)	Observational study	PUBMED query
(Bowman et al., 2019)	Trial not completed	Citation searching

Table S3. For positive brain imaging results from studies where cognition is evaluated, number of results positive on cognition.

	Results from controlled studies + (C)		Results from studies with no control group + (NC)	
	+ (Cog)	- (Cog)	+ (Cog)	- (Cog)
**PE**				
nCI (N=0, k=0)	0	0	0	0
CI (N=7, k=7)	2 (67%)	1 (33%)	4 (100%)	0 (0%)
**Nutrition**				
nCI (N=0, k=0)	0	0	0	0
CI (N=5, k=5)	4 (80%)	1 (20.0%)	0	0
**CT**				
nCI (N=2, k=2)	1 (50%)	1 (50%)	0	0
CI (N=8, k=12)	3 (60%)	2 (40%)	7 (100%)	0 (0%)
**MD**				
nCI (N=1, k=1)	1 (100%)	0 (0%)	0	0
CI (N=4, k=5)	2 (50%)	2 (50%)	1 (100%)	0 (0%)

Were not included in this tables results from intervention intermediate timepoints (Delrieu et al., 2020 : results at 6 months, Soininen et al., 2017) or followup measures (Broadhouse et al., 2020 : results at 18 months), from a mixed sample of participants (Neth et al., 2020 : ketone uptake and glucose metabolism), and studies reporting only results from correlation analyses (Anderson-Hanley et al., 2018; Hama et al., 2020; Vermeij et al., 2016), or analyzing specific subgroups within a clinical trial (Jernerén et al., 2015; Kaufman et al., 2021).

Abbreviations: + (C)=studies with a control condition and positive results on imaging data, + (Cog)=studies with positive results on cognition, - (Cog)=studies with negative results on cognition, + (NC)=studies with no control condition and positive results on imaging data, CI=Cognitive Impairment, CT=Cognitive Training, k=Number of results, MD=Multidomain, N=Number of studies, nCI=No Cognitive Impairment, PE=Physical Exercise.

2 FIGURES

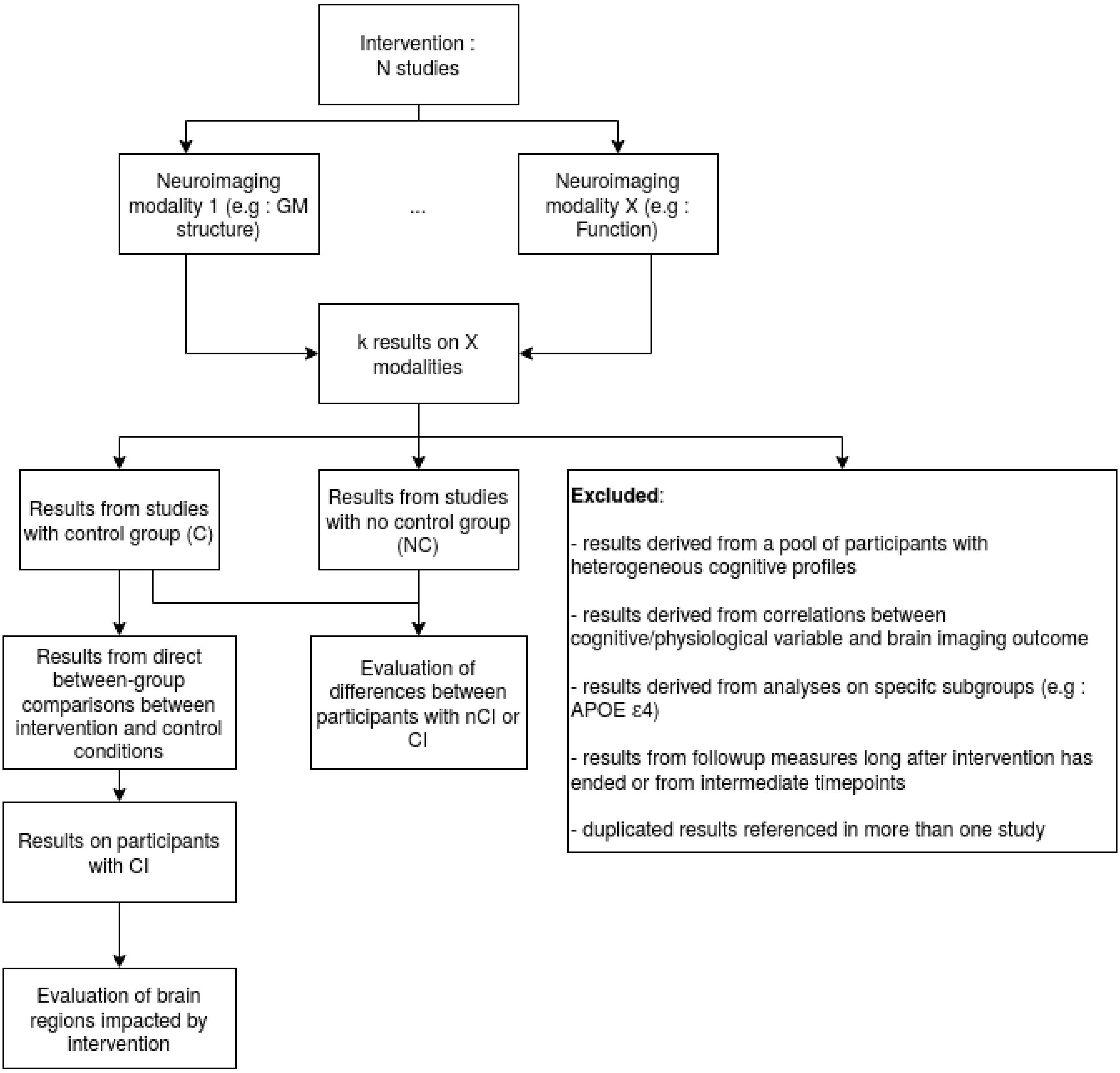

Figure S1. Selection of results used to assess differences between participants' cognitive profiles and effect of intervention on brain regions. The effect of interventions was assessed on multiple neuroimaging modalities : GM structure, function, WM structure, metabolism (glucose and other metabolites), perfusion, ultrasound and brain amyloid imaging.
Abbreviations : nCI=no cognitive impairment, APOE= APOE=Apolipoprotein E, CI=cognitive impairment, GM=Gray Matter, WM=White Matter.

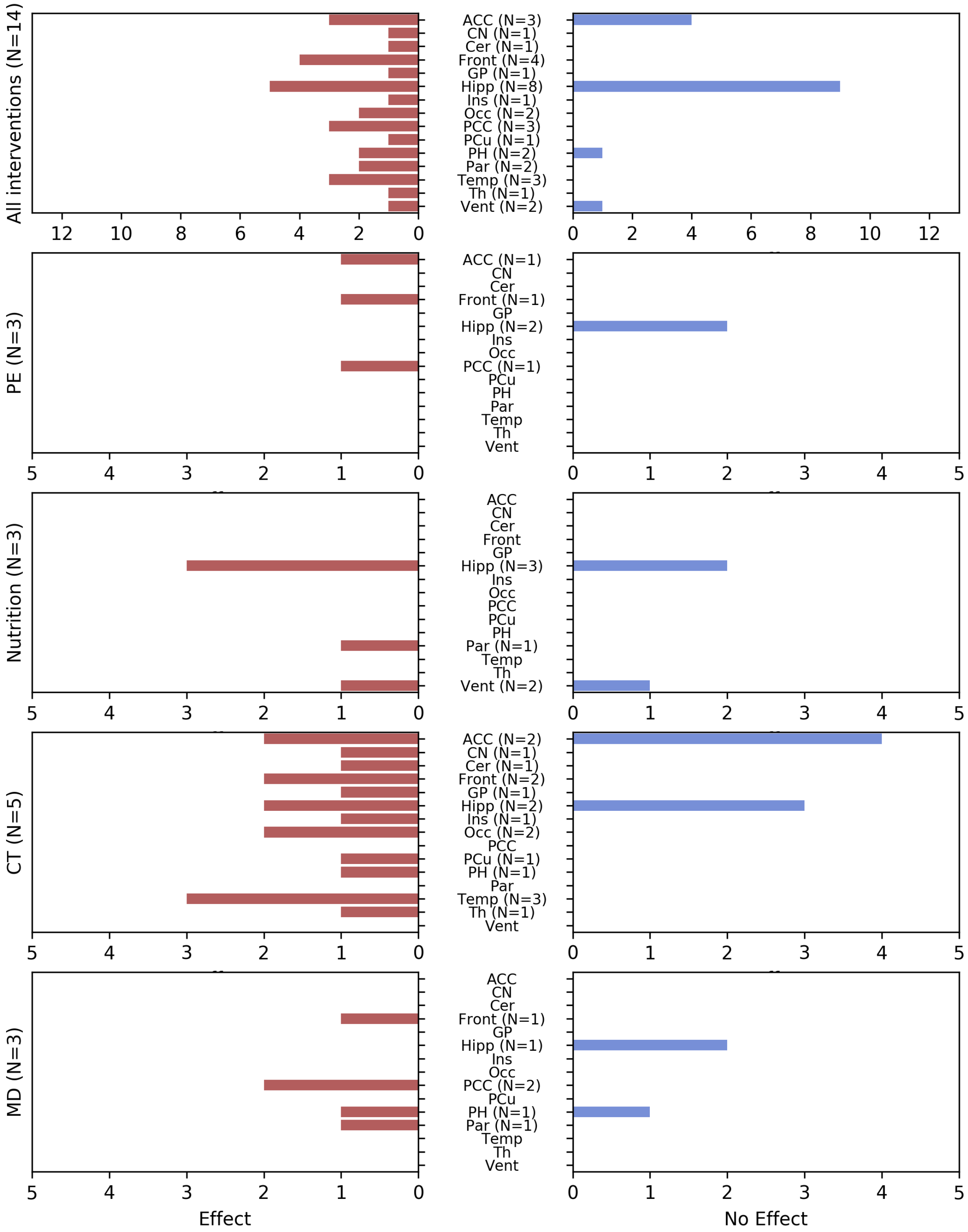

Figure S2. Brain regions affected by the interventions for participants with CI, for all imaging modalities and after direct comparison to a sham intervention.
The effect of an intervention on a region (positive result, red) can be detected after a whole brain or target-region analysis. The lack of effect of an intervention on a region (null result, blue) is reported only if the region has been specifically targeted in the study's analysis. Abbreviations : ACC=Anterior Cingulate Cortex, Cer=Cerebellum, CN=Caudate nuclei, CT=Cognitive Training, Front=Frontal area, GP=Globus Pallidus, Hipp=Hippocampus, Ins=Insula, MD=Multidomain, N=Number of studies; Occ=Occipital areas, PCC= Posterior Cingulate Cortex, PCu=Precuneus, PE=Physical Exercise, PH=Parahippocampal area, Par=Parietal areas, Temp=Temporal areas, Th=Thalamus, Vent=Ventricles.

REFERENCES

Bowman, G. L., Silbert, L. C., Dodge, H. H., Lahna, D., Hagen, K., Murchison, C. F., et al. (2019). Randomized Trial of Marine n-3 Polyunsaturated Fatty Acids for the Prevention of Cerebral Small Vessel Disease and Inflammation in Aging (PUFA Trial): Rationale, Design and Baseline Results. *Nutrients* 11, E735. doi:10.3390/nu11040735

Doniger, G. M., Beeri, M. S., Bahar-Fuchs, A., Gottlieb, A., Tkachov, A., Kenan, H., et al. (2018). Virtual reality-based cognitive-motor training for middle-aged adults at high Alzheimer's disease risk: A randomized controlled trial. *Alzheimers Dement (N Y)* 4, 118–129. doi:10.1016/j.trci.2018.02.005

Hsu, C. L., Best, J. R., Davis, J. C., Nagamatsu, L. S., Wang, S., Boyd, L. A., et al. (2018). Aerobic exercise promotes executive functions and impacts functional neural activity among older adults with vascular cognitive impairment. *Br J Sports Med* 52, 184–191. doi:10.1136/bjsports-2016-096846

Pantoni, L., Poggesi, A., Diciotti, S., Valenti, R., Orsolini, S., Della Rocca, E., et al. (2017). Effect of Attention Training in Mild Cognitive Impairment Patients with Subcortical Vascular Changes: The RehAtt Study. *J Alzheimers Dis* 60, 615–624. doi:10.3233/JAD-170428

Qi, M., Zhu, Y., Zhang, L., Wu, T., and Wang, J. (2018). The effect of aerobic dance intervention on brain spontaneous activity in older adults with mild cognitive impairment: A resting-state functional MRI study. *Exp Ther Med* doi:10.3892/etm.2018.7006

Sala-Vila, A., Arenaza-Urquijo, E. M., Sánchez-Benavides, G., Suárez-Calvet, M., Milà-Alomà, M., Grau-Rivera, O., et al. (2021). DHA intake relates to better cerebrovascular and neurodegeneration neuroimaging phenotypes in middle-aged adults at increased genetic risk of Alzheimer disease. *Am J Clin Nutr* 113, 1627–1635. doi:10.1093/ajcn/nqab016

Smart, C. M., Segalowitz, S. J., Mulligan, B. P., Koudys, J., and Gawryluk, J. R. (2016). Mindfulness Training for Older Adults with Subjective Cognitive Decline: Results from a Pilot Randomized Controlled Trial. *JAD* 52, 757–774. doi:10.3233/JAD-150992

Tang, Y., Xing, Y., Zhu, Z., He, Y., Li, F., Yang, J., et al. (2019). The effects of 7-week cognitive training in patients with vascular cognitive impairment, no dementia (the Cog-VACCINE study): A randomized controlled trial. *Alzheimers Dement* 15, 605–614. doi:10.1016/j.jalz.2019.01.009

Um, Y. H., Wang, S.-M., Kim, N.-Y., Kang, D. W., Na, H.-R., Lee, C. U., et al. (2020). Effects of Moderate Intensity Exercise on the Cortical Thickness and Subcortical Volumes of Preclinical Alzheimer's Disease Patients: A Pilot Study. *Psychiatry Investig* 17, 613–619. doi:10.30773/pi.2020.0214

Xia, R., Qiu, P., Lin, H., Ye, B., Wan, M., Li, M., et al. (2019). The Effect of Traditional Chinese Mind-Body Exercise (Baduanjin) and Brisk Walking on the Dorsal Attention Network in Older Adults With Mild Cognitive Impairment. *Front Psychol* 10, 2075. doi:10.3389/fpsyg.2019.02075
